# Core lipid, surface lipid and apolipoprotein composition analysis of lipoprotein particles as a function of particle size in one workflow integrating asymmetric flow field-flow fractionation and liquid chromatography-tandem mass spectrometry

**DOI:** 10.1371/journal.pone.0194797

**Published:** 2018-04-10

**Authors:** Zsuzsanna Kuklenyik, Jeffery I. Jones, Michael S. Gardner, David M. Schieltz, Bryan A. Parks, Christopher A. Toth, Jon C. Rees, Michael L. Andrews, Kayla Carter, Antony K. Lehtikoski, Lisa G. McWilliams, Yulanda M. Williamson, Kevin P. Bierbaum, James L. Pirkle, John R. Barr

**Affiliations:** Clinical Chemistry Branch, Division of Laboratory Sciences, Centers for Disease Control and Prevention, Atlanta, Georgia, United States of America; Centre de Recherche en Cancerologie de Lyon, FRANCE

## Abstract

Lipoproteins are complex molecular assemblies that are key participants in the intricate cascade of extracellular lipid metabolism with important consequences in the formation of atherosclerotic lesions and the development of cardiovascular disease. Multiplexed mass spectrometry (MS) techniques have substantially improved the ability to characterize the composition of lipoproteins. However, these advanced MS techniques are limited by traditional pre-analytical fractionation techniques that compromise the structural integrity of lipoprotein particles during separation from serum or plasma. In this work, we applied a highly effective and gentle hydrodynamic size based fractionation technique, asymmetric flow field-flow fractionation (AF4), and integrated it into a comprehensive tandem mass spectrometry based workflow that was used for the measurement of apolipoproteins (apos A-I, A-II, A-IV, B, C-I, C-II, C-III and E), free cholesterol (FC), cholesterol esters (CE), triglycerides (TG), and phospholipids (PL) (phosphatidylcholine (PC), sphingomyelin (SM), phosphatidylethanolamine (PE), phosphatidylinositol (PI) and lysophosphatidylcholine (LPC)). Hydrodynamic size in each of 40 size fractions separated by AF4 was measured by dynamic light scattering. Measuring all major lipids and apolipoproteins in each size fraction and in the whole serum, using total of 0.1 ml, allowed the volumetric calculation of lipoprotein particle numbers and expression of composition in molar analyte per particle number ratios. Measurements in 110 serum samples showed substantive differences between size fractions of HDL and LDL. Lipoprotein composition within size fractions was expressed in molar ratios of analytes (A-I/A-II, C-II/C-I, C-II/C-III. E/C-III, FC/PL, SM/PL, PE/PL, and PI/PL), showing differences in sample categories with combinations of normal and high levels of Total-C and/or Total-TG. The agreement with previous studies indirectly validates the AF4-LC-MS/MS approach and demonstrates the potential of this workflow for characterization of lipoprotein composition in clinical studies using small volumes of archived frozen samples.

## Introduction

Lipoproteins (Lps) are complex molecular assemblies that are key participants in the intricate cascade of extracellular lipid metabolism with important consequences in the formation of atherosclerotic lesions and the development of cardiovascular disease (CVD) [1–4]. The surface of Lps contain amphipathic phospholipids (PL), with free un-esterified cholesterol (FC) stabilizing their assembly into monolayers where the hydrophobic fatty acyl carbon chain of the PL molecules enclose a non-polar lipid core, consisting of mainly cholesterol esters (CE) and triglycerides (TG). The polar PL head groups face the aqueous plasma environment and provide a surface for non-covalent binding of apolipoproteins (apos), proteins with lipid binding amphipathic α-helices and β-sheets [5, 6].

The two apos that are most essential in normal lipid metabolism are apoA-I and apoB (apoB-100 and its truncated form apoB-48). Because of their importance to the biogenesis and structural integrity of Lps, apoA-I and apoB containing Lps are designated as LpA and LpB families [7]. During extracellular circulation, LpA and LpB particles change their lipid composition through enzymatic and molecular transfer processes modulated by the numerous exchangeable apos, including apos A-II, A-IV, C-I, C-II, C-III and E. The binding of exchangeable apos is influenced by the PL composition of the Lp surface, including phosphatidylcholines (PC) and sphingomyelins (SM) along with less abundant classes, phosphatidylethanolamines (PE), phosphatidylinositols (PI), and lysophosphatidylcholines (LPC) [8].

Lps can be physically separated by exploiting their differences in intrinsic hydrodynamic diameter, diffusion coefficient, sedimentation coefficient, and electrophoretic mobility. Traditionally, the chosen physicochemical property for the classification of Lps was density; into high, low, intermediate and very low density Lp classes (HDL, LDL, IDL and VLDL), and chylomicrons. To some degree, the density of Lps are the reflection of the size and qualitative differences in composition of Lps; smaller core volume (CE+TG) corresponds with greater protein/lipid ratios and therefore higher density. This loose correspondence of increasing Lp size, decreasing Lp density, relative electrophoretic mobility, and apoA-I vs. apoB-100 content led to a convenient framework for studying the metabolic functions of Lps.

However, within HDL, LDL, IDL, and VLDL classes, Lps are heterogeneous and vary in protein/lipid composition, i.e. “lipoproteome” [9]. These Lp sub-classes can be differentiated by using tube gel electrophoresis, gradient gel electrophoresis, sequential ultracentrifugation, and nuclear magnetic resonance (NMR). However, studies that tried to correlate the results of these different techniques showed only moderate agreement [10–12]. Harmonization of sub-fractionation methods is inherently challenging because they are based on different physicochemical properties, such as density and size that are not directly interchangeable. In addition the different methods rely on different detection techniques and signal deconvolution algorithms. The essential characteristic that could bring Lp sub-fractionation methods on a common base is lipid and protein composition of sub-classes which can be most directly linked to their corresponding metabolic functions. Although a “lipoproteome” composition based conceptual framework of Lps and the measurement of an extended panel of lipid and protein constituents has been advocated for many years [13–15], the development of high throughput multiplexed mass spectrometry (MS) based analytical techniques has allowed comprehensive Lp analysis to become a reality only recently [16–18].

Composition analysis of Lp sub-classes is challenging because of the potential for commonly used sub-fractionation techniques to introduce modifications in the native composition of Lps due to intense centrifugal forces during ultracentrifugation, squeezing into pores during gradient electrophoresis, dilution or interaction with the column packing material during size exclusion chromatography. An alternative preparative sub-fractionation technique is asymmetric flow field-flow fractionation (AF4). The application of AF4 for separation of Lps and its advantage over other sizing techniques is well demonstrated [19–22]. Unfortunately, AF4 is still considered a non-conventional technique for the separation of Lps in lipid metabolism research.

AF4 is a gentle size separation technique where the differentiating medium is parallel layers of laminar flow inside a thin channel. The intact Lp particles are eluted by the carrier fluid at physiological salt concentration in the order of their diffusion coefficient. The retention time is fundamentally related to hydrodynamic particle size based on the Stokes-Einstein equation. The elution time and size resolution is determined by the channel thickness, and a cross flow field which is perpendicular to the channel flow and created by withdrawal of the carrier buffer through one of the channel walls that is made of a porous ceramic block covered by a molecular weight cut-off membrane. The strength of the cross flow field is determined by the ratio of the cross flow to the channel flow. Proteins and other serum components that are smaller than the molecular weight cutoff are carried across the membrane into waste while components above the molecular weight cutoff are eluted at the end of the channel and carried through the detector into the fraction collector. An important advantage of the AF4 technique is that the particles remain concentrated in close vicinity of the membrane covered wall during the separation, within ~10% of the channel thickness, without dilution into the entire volume of the carrier buffer passing through the channel. The particles from the laminar layers near the wall can be also collected separately from the main part of the carrier buffer flow. Splitting the total channel flow in a ratio as low as 1:10 allows the collection of relatively concentrated fractions.

As metabolically functional entities Lps are nanoscale particles. Characterization of Lp particle composition, i.e. number of molecules of individual protein and lipid constituents per Lp particle, requires measurement of particle concentration or particle number (Lp-P), in moles of particle per volume of serum. Lp-P can be directly determined by ion mobility separation and light scattering detection of particles [23], but it has to be preceded by removal of other non-lipidated proteins of similar size by ultracentrifugation and manual removal of the Lp density layers. In the case of LpB particles, an alternative to Lp-P measurement is apoB concentration measurement. Based on understanding of the biogenesis of LpB particles (LDL and vLDL) and evidence from immunolabeling experiments, it is generally accepted that there is one apoB-48 or apoB-100 in each LpB particle [24, 25]. With this assumption, LpB particle numbers in serum can be directly determined based on the total apoB concentration. In the case of non-apoB containing particles, the number of all protein molecules per particle, including apoA-I in HDL, vary with particle size, and there is no specific protein constituent concentration which can be directly converted into HDL-P [26].

An alternative approach to Lp-P measurement and compositional analysis is based on preparative fractionation by size, followed by measurement of particle size and concentration of all major lipid/protein constituents in each fraction. This approach is called the volumetric approach [26]. When the size separation and analysis is performed in sufficiently small size increments, Lp-P in each fraction can be determined by

$$Lp-P\;by\;\text{fraction}=\frac{\sum \left(\left[\text{Molar Analyte Concentration}\right]\text{*}\left[\text{Theoretical Molecular Volume}\right]\right)}{\text{Volume of 1 mole of Particles}}[mole/L]$$ (1)

The average stoichiometric composition of the particles can be derived by

$$\left[\text{Number of analyte molecules per}\;1\;\text{particle}\right]=\frac{\left[\text{Analyte concentration in serum by fraction}\right]}{\left[\text{Lp}-\text{P by fraction}\right]}[mole/mole]$$ (2)

The volumetric approach based on preparative fractionation and measurement of all main lipid and protein constituents has been previously demonstrated on small numbers of samples or sample pools [26–28]. Analysis of a greater number of samples by these approaches was limited due to single-analyte immunoaffinity and enzymatic/colorimetric assays that were available at the time, and by the requirement for starting with milliliters of fresh samples. In recent years, mass spectrometry (MS) based, sensitive, multiplexed and quantitative proteomics and lipidomics methods have been developed. [29–32]. Using MS analysis of all main lipids and proteins in Lp fractions, the volumetric approach can be applied in a straightforward and high throughput manner, and requires a much smaller amount of each specimen.

In this report using a previously peer reviewed AF4 method [33], we demonstrate that AF4 allows separation of both HDL and LDL sub-classes simultaneously with sufficient size resolution that is applicable for showing compositional differences between Lp sub-classes by liquid chromatography and tandem mass spectrometry (LC-MS/MS) analysis. In each collected size fraction, we measured the average hydrodynamic size by dynamic light scattering (DLS), and applied three validated high throughput, multiplexed LC-MS/MS methods to quantify non-polar lipids (FC, CE, TG) [34], polar lipids (PC, SM, LPC, PE and PI) and a panel of apolipoproteins (A-I, A-II, A-IV, B-100, C-I, C-II, C-III and E) [35]. Applying this workflow, we were able to acquire both fractionated and total serum measurements for each target constituent for 110 serum samples with wide range of Total-C and Total-TG levels, using a total of 100 μL from each sample. In this report, we evaluate the accuracy of the approach by volumetric determination of Lp-P and particle composition expressed in number of analyte molecules per particle, showing composition differences both between Lp classes (i.e., HDL, LDL) and between size fractions within Lp classes. By calculation of physical characteristics such as surface thickness and core diameter and surface/core volume ratio as well as lipid/lipid and apo/apo molar ratios, we show composition differences between sample categories with normal and high Total-C and/or Total-TG levels. By comparison of the results with observations of past investigations we indirectly validate our AF4-LC-MS/MS based composition analysis approach.

## Materials and methods

### Serum samples

Units of fresh human serum from 4 individuals were purchased from Interstate Blood Bank (Memphis, TN, U.S.A.) and mixed together using equal volumes for the preparation of calibrator pools and quality control (QC) pools. After mixing, the pool was distributed into 1 mL aliquots and stored at -80°C. Lipoprotein density fractions were obtained from Intracel Resources (Frederick, MD, USA). Frozen serum samples from 110 fasting individuals were purchased from BioreclamationIVT (NY, USA) (Protocol Number 2010–017), and stored at -80°C until analysis. The health status or demographics of the donors were not available. Ethics statement: All samples were handled anonymously. The project was approved as research not involving identifiable human subjects under U. S. Health and Human Services Department Policy for Protection of Human Research Subjects codified in the Code of Federal Regulations at 45 CFR part 46.

### Size fractionation by asymmetric flow-field flow fractionation (AF4)

**Fig 1:**
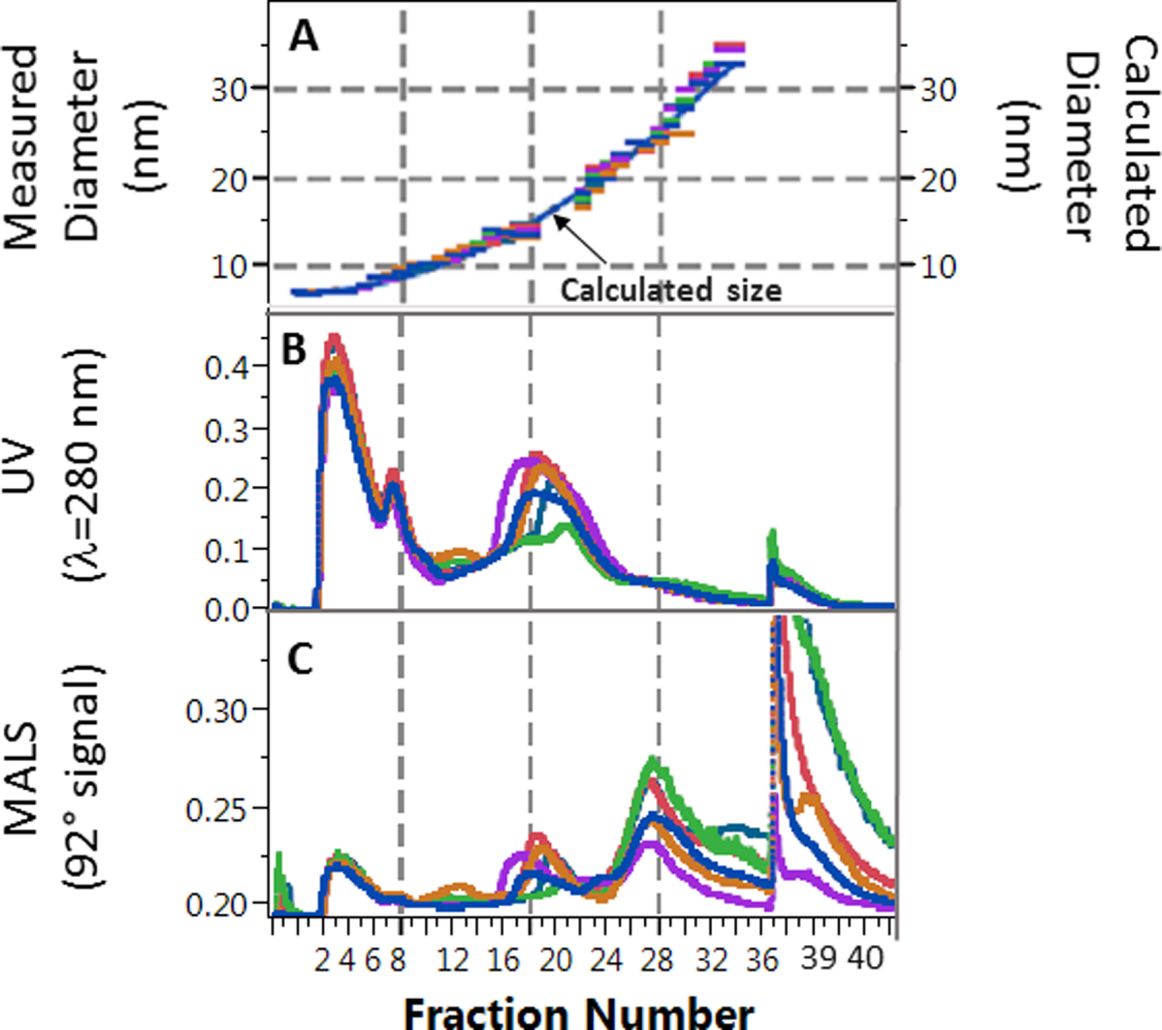
Overlay of data for six serum samples from a representative asymmetric flow field-flow fractionation run. A: Overlay of average hydrodynamic diameter measured by dynamic light scattering vs. fraction number. Continuous line shows calculated size based on quadratic fit. B: Overlay of corresponding UV signal during fractionation. C: Overlay of 92° signal from the multi-angle light scattering (MALS) detector during fractionation. Grid lines are shown for ease of visualization of fraction numbers across graphs.

Details of the optimization of our AF4 separation method was described previously [33]. The AF4 system (AF2000, PostNova Analytics, Salt-Lake City, USA) was used with 10 mM sodium bicarbonate and 150 mM sodium chloride in deionized water (pH 7.4) as carrier fluid with 0.1 mL/min outlet flow (total channel flow was 0.45 mL/min, split into 0.1 mL/min detector flow and 0.35 mL/min slot flow). The autosampler was kept at 4°C temperature. The AF4 method was optimized to separate 50 μL serum into 40 size fractions. The injection/focusing time was 12 min with 0.2 mL/min inlet flow. The gradient cross flow (F) was programed with an exponential decay of F(t) = F_start_-(F_start_-F_end_)*(t/t_end_)^0.8^ (t_end_ = 90 min, F_start_ = 3.5 mL/min, and F_end_ = 0 mL/min). After 90 min the crossflow was turned off allowing complete elution of all remaining particles for an additional 20 min. The fraction collection started with a 5 min delay after injection/focusing, then collecting 38 fractions with 2.5 min time increments (5–95 min), and 2 more fractions with 4 min increments. During the fractionation, the elution of the serum components was monitored by UV and multi-angle light scattering (MALS) detectors (Fig 1). The fractions were collected into deep-well 96-well Eppendorf plates, using an Agilent 1200 analytical fraction collector kept at 4°C. The flow rate was recorded during the entire AF4 elution run using a microfluidic flow meter installed between the outlet of the MALS detector and the fraction collector inlet (Elveflow, France). The volumes of the fractions were determined from the fraction collection time increment and the corresponding measured flow rates. The volume of the fractions were used for the calculation of the amount of analytes in each fraction and the calculation of AF4 channel recovery.

### Hydrodynamic size measurement by dynamic light scattering

Dynamic light scattering (DLS) is a “first principle” size measurement technique, measuring fluctuations in light intensity as deflected from the surface of particles in Brownian motion. As long as the particles are homogeneous in size, the fluctuation in the scattered light intensity can be directly transformed into diffusion coefficient and hydrodynamic size information without calibration using purely mathematical calculations built into the DLS instrument software. The accuracy of the DLS size measurements was confirmed by measurements of certified 20–100 nm NIST (National Institute of Standards and Technology) Traceable Particle Size Standards (Thermo Scientific, USA). A 10 μL aliquot of each fraction was transferred into a Corning clear-bottom 384-well plate (Sigma-Aldrich, USA). The average hydrodynamic size in each AF4 fraction was determined using a Dynapro DLS plate reader (Wyatt Technologies, Santa Barbara, USA). A typical size vs. fraction number graph is shown in Fig 1A. The hydrodynamic size vs. fraction number calibration curve was constructed by fitting one quadratic function on the DLS data for each AF4 batch. The hydrodynamic size in each fraction was calculated by substituting the corresponding fraction numbers into the quadratic function. Thus, even with some batch-to-batch shifts of the AF4 retention times (corresponding with a shift of 1–3 fractions), the data from multiple batches could be compiled on a common hydrodynamic size scale.

### LC-MS/MS analysis of fractions and diluted serum samples

The optimization and validation of our LC-MS/MS based methods are described in other publications (method details provided in S1 File). For the LC-MS/MS analysis of FC, CE and TG [34], a simplified one-pot lipid extraction protocol was used without ester hydrolysis: precipitation with 200 μL ethanol spiked with isotope labeled internal standards, followed by evaporation and then extraction of the dry pellet with nonane. Six μL of the extracts were injected directly from the same 96-well plate; the extracted pellet remained on the bottom of each well. Reproducible in-source nitrogen collision-induced dissociation (CID) using a 4000 Qtrap mass spectrometer (Sciex, Framingham, MA) was achieved with the use of optimal cone voltage (declustering potential), generating FC, CE and TG specific precursor fragment ions for multiple reaction monitoring (MRM). The quantitation and one confirmation ion transitions were specific to all CE and TG species as lipid classes regardless of differences in alkyl chain length and saturation of individual CE and TG molecules. Representative chromatograms are shown in S1 Fig. A dilution series of pooled serum provided by NIST was used for calibration. The method accuracy was assessed with analysis of 5 inter-laboratory standardization materials provided by the Lipid Standardization Program of the Centers for Disease Control and Prevention (CDC), showing 4% bias for Total-C and 5% for Total-TG. Repeated analysis of a quality control pool showed inter-day CV of 8–10% (N = 17) (S1 Table).

For the LC-MS/MS quantification of PC, SM, PE, LPC and PI (as lipid classes), a targeted lipidomics approach was used [36] with a modified high throughput extraction protocol in 96-well plate format (method details provided in S2 File). The extraction protocols included protein precipitation by 200 μL ethanol spiked with one isotope labeled internal standard for each lipid class; followed by evaporation to dry pellet and extraction with a mix of water (2%), isopropanol (43%) and nonane (55%). Five μL of the extracts were injected directly from the same 96-well plate. An Acquity UHPLC system (Waters, USA) was used with a 6500 Qtrap mass spectrometer (Sciex, Framingham, MA) operated with a TurboSpray IonDrive source (ESI). MRM transitions of 15 PI species were monitored in negative ion mode. The other PL classes were monitored in positive ion mode; 19 PC species, 18 SM species, 18 PE species, and 7 LPC species with various alkyl chain length and saturation. Total ion chromatograms for each PL class were generated by summing corresponding individual species signals. A typical total ion chromatogram is shown in S2 Fig. A serum pool was value assigned using purified PL species as calibrators purchased from Avanti Polar Lipids (USA). The reproducibility of the concentration measurements by PL classes was assessed with repeated measurements of a 1:100 diluted pool, showing inter-day CV of 6–10% (N = 17) (S1 Table).

The description and validation of the on-column trypsin digestion coupled LC-MS/MS protein analysis method is reported in previous publications [35, 37] and in S3 File. The workstation included an LC system (Perfinity Biosciences, USA) and a 6500 QTRAP mass spectrometer (Sciex, Framingham, MA). A dilution series of a value assigned serum pool was used as a calibrator. The serum pool was value assigned for apos A-II, A-IV, C-I, C-II, C-III and E by standard addition methodology using purified protein standards. The value assignment for apoA-I and apoB-100 was obtained using a validated in-solution trypsin digestion method with proteotypic peptide standards [38]. The accuracy of the value assignment was assured by amino acid analysis of the protein and peptide standards, and repeated analysis of World Health Organization (WHO) harmonization standards of apoA-I (SP1-01) and apoB-100 (SP3-08) [35, 38]. Method precision based on repeated analysis of 1:100 diluted pooled sera showed inter-day CV of 12–25% (N = 76) (S1 Table).

### Workflow and quality control procedures

**Fig 2:**
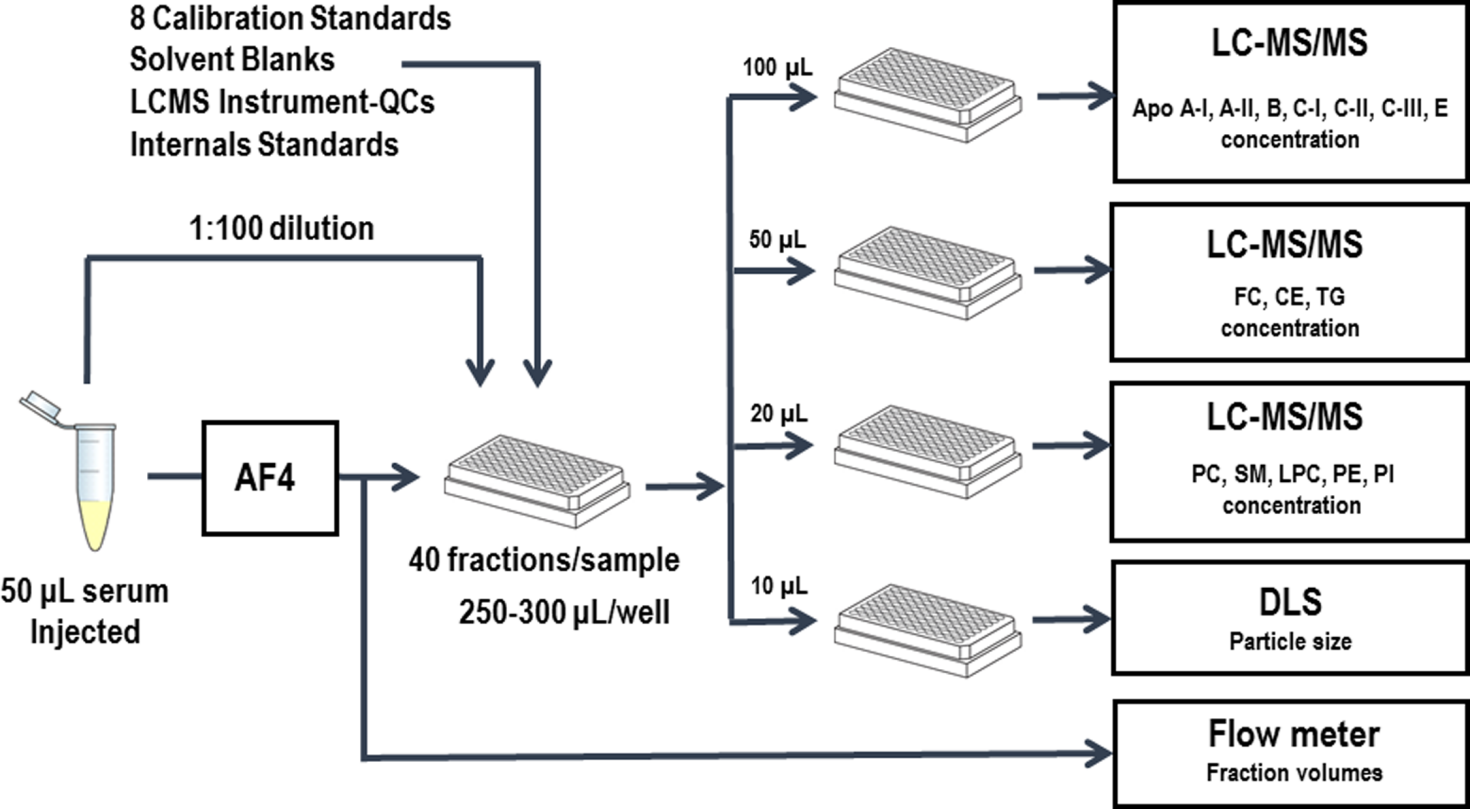
Schematic presentation of the workflow. AF4: asymmetric flow field-flow fractionation; DLS: dynamic light scattering; LC-MS/MS: liquid chromatography-tandem mass spectrometry.

The reproducibility of the AF4 fractionation was monitored by including the fractionation of a quality control serum pool (AF4-QC) into each AF4 sample batch. A typical batch analysis included the AF4 separation of 6 samples, the AF4-QC and 5 unknown samples (Fig 2). Samples were pre-categorized by the vendor (BioreclamationIVT). Each batch of AF4-LC-MS/MS runs included samples from each of the sample categories to minimize between-run bias in the data analysis. From 50 μL of serum injected into the AF4 system, a set of 40 fractions from each sample was collected. After the AF4 run, 10 μL aliquots from each fraction was transferred into a 384-well plate for size measurements by DLS.

A 10 μL serum aliquot from each of the original autosampler vials was diluted 1:100 with AF4 buffer. From each of these diluted serum samples, one 300 μL aliquot was added on the corresponding fraction plates. For each set of 40 fractions and diluted serum aliquots per serum sample, 50 μL, 20 μL, and 100 μL aliquots were transferred into respective shallow 0.5-mL 96-well plates. These aliquots were used for non-polar lipid, polar lipid, and protein analysis, respectively.

Before analysis on the various LC-MS/MS workstations, empty wells were filled with two aliquots of a solvent blank and two aliquots of an instrument test sample (LCMS-QC), a serum pool diluted 1:100 with AF4 buffer. The remaining 8 wells were filled with 100 μL aliquots of the dilution series of the calibrator serum pool. A typical plate layout is shown in S4 Fig. On each instrument, the LCMS-QC was injected twice at the beginning and at the end of the instrument run. The solvent blank was injected twice before the lowest calibrator. The 1:100 diluted AF4-QC and unknown whole serum samples were injected in triplicate.

The workflow schedule of a typical 6-sample batch included the AF4 fractionation for 12 hours overnight. Distribution of aliquots into analysis plates were done the next morning for hydrodynamic size measurements, followed immediately by lipid extractions and reagent additions, collectively taking 1–2 hours each day. The LC-MS/MS analysis required a minimum of five workstations: one workstation for running 3 plates for non-polar lipid analysis, one workstation for running 3 plates for polar lipid analysis, and three workstations, each running one of three plates for protein analysis.

### Calculations

Each AF4 fraction was treated as a unique Lp particle type or species with a unique particle number (Lp-P) expressed in units of nmol/L in serum. The analyte concentrations in each fraction were multiplied with the measured actual volume of each fraction, and divided by the volume of the serum injected into the AF4 system, yielding analyte concentrations in serum by fraction, as shown in Eq 3.

$$\left[\text{Analyte concentration in serum by fraction}\right]=\frac{\left[\text{Concentration of analyte in fraction}\right]\text{*}\left[\text{Fraction Volume}\right]}{\left[\text{Volume of serum injected into the AF}4\;\text{ channel}\right]}*\frac{1}{\text{AF}4_{\text{Recovery}}}[mole/L]$$ (3)

The AF4 channel recovery for each analyte was calculated from the sum of all fraction concentrations divided by the total concentration measured in the unfractionated whole serum (Eq 4).

$$\text{AF}4\_\text{Recovery}=\frac{\sum_{1}^{40}\left[\text{Concentration of analyte in fraction}\right]\text{*}\left[\text{Fraction Volume}\right]}{\left[\text{Total concentration in serum}\right]\text{*}\left[\text{volume of serum injected into the AF}4\;\text{channel}\right]}$$ (4)

We adapted a similar volumetric Lp-P calculation scheme used in previous studies by Segrest, McNamara, and Teerlink [26–28]. Total particle core volumes and surface volumes in each fraction were calculated by multiplying the [analyte concentrations in serum by fraction] (Eq 3) with the literature reported molecular volumes of FC (V_FC_ = 0.610 nm^3^), CEs (V_CE_ = 1.179 nm^3^), TGs (V_TG_ = 1.575 nm^3^), and PLs (V_PL_ = 1.307 nm^3^). Protein volumes were calculated from the molecular weight of each protein (MW_protein_) multiplied with partial specific volume of 1.212 nm^3^/kDa (*v*_protein_) (Eq 5) [39].

$$\frac{\text{Total Particle Core Volume by fraction}}{\text{L of serum}}=({\text{V}}_{\text{CE}}\text{*}\left[\text{CE}\right]\text{*}N_{A})+({\text{V}}_{\text{TG}}\text{*}\left[\text{TG}\right]\text{*}N_{A})+({\text{V}}_{\text{FC}}\text{*}N_{A}\text{*}\left[{\text{FC}}_{\text{Core}}\right])\;\left[{\text{nm}}^{3}/\text{L}\right]$$ (5)

$$\frac{\text{Total Particle Surface Volume by fraction}}{\text{L of serum}}=({\text{V}}_{\text{PL}}\text{*}\left[\text{PL}\right]\text{*}N_{A})+({\text{V}}_{\text{FC}}\text{*}\left[{\text{FC}}_{\text{surface}}\right]\text{*}N_{A})+(\sum ({\text{MW}}_{\text{Protein}}\text{*}v_{\text{Protein}}\text{*}\left[\text{Protein}\right]\text{*}N_{A}))\;\left[{\text{nm}}^{3}/\text{L}\right]$$ (6)

Eq 6 shows the calculation for total particle surface volume by fraction per liter of serum, where N_A_ is Avogadro’s number [mole^-1^]. [FC_core_] and [FC_surface_] were calculated by assuming a partitioning coefficient of 5 (5/6 *vs*. 1/6) between polar and non-polar lipid phases [27] (Eqs 7 and 8, respectively).

$$\left[{\text{FC}}_{\text{core}}\right]=\frac{1}{6}*\left[\text{FC}\right]*\frac{\left[\text{CE}\right]+\left[\text{TG}\right]}{\left[PL\right]}\;\left[\text{mole}/\text{L}\right]$$ (7)

$$\left[{\text{FC}}_{\text{surface}}\right]=\left[\text{FC}\right]*\left(1-\frac{1}{6}\frac{\left[\text{CE}\right]+\left[\text{TG}\right]}{\left[PL\right]}\right)\;\left[\text{mole}/\text{L}\right]$$ (8)

The dehydrated spherical particle volumes by fraction were calculated by using the average hydrodynamic diameter in the fractions measured by DLS (d_DLS_), and subtracting 1 hydration water layer (n), with thickness of w_h_ = 0.3 nm around each particle (Eq 9).

$$\text{Dehydrated Volume of}\;1\;\text{mole of particles}=N_{A}*\frac{4\pi }{3}\text{*}{\left(\frac{{\text{d}}_{\text{DLS}}-2{\text{*n*w}}_{\text{h}}}{2}\right)}^{3}\left[\text{mole}/\text{L}\right]$$ (9)

Therefore, the number of particles in the fraction can be calculated using Eq 10.

$$\text{Lp}-\text{P by fraction}=\frac{\frac{\text{Total Particle Core Volume by Fraction}}{\text{L of serum}}+\frac{\text{Total Particle Surface Volume by Fraction}}{\text{L of serum}}}{\text{Dehydrated Volume of }\;1\;\text{mole of particles}}\left[\text{mole}/\text{L}\right]$$ (10)

The number of analyte molecule per particle can be also expressed in Eqs 11 and 12.

$$\frac{\text{analyte}}{\text{Lp}-\text{P}}=\frac{\left[\text{Analyte Concentration in Serum by Fraction}\right]}{\text{Lp}-\text{P}}\;\left[\text{mole}/\text{mole}\right]$$ (11)

$$\frac{\text{analyte}}{\text{Lp}-\text{P}}=N_{A}*\frac{4\pi }{3}\text{*}{\left(\frac{{\text{d}}_{\text{DLS}}-2{\text{*n*w}}_{\text{h}}}{2}\right)}^{3}\text{*}\frac{\left[\text{Analyte Concentration in Serum by Fraction}\right]}{\frac{\text{Total Particle Core Volume by Fraction}}{\text{L of serum}}+\frac{\text{Total Particle Surface Volume by Fraction}}{\text{L of serum}}}\;\left[\text{mole}/\text{mole}\right]$$ (12)

### Data analysis

To convert the size information from a continuous to a uniform quantized scale, the size measurements were binned into 1 nm, 2 nm and 3 nm integer ranges. The absolute serum concentrations and analytes/Lp-P molar ratios were calculated by summing and averaging in each size bin. In the <18 nm size region, binning by size meant using 2–3 fractions per 1 nm increment, in the 18–30 size region 2–3 fractions per 2 nm increment, and in the >30 m, region 2–3 fractions per 3 nm increment. Mean comparison between size bins and between sample categories within size bins were evaluated using pair-wise Student’s t test. Differences with two-tailed p<0.05 were considered significant. Pairwise linear correlations were evaluated based on Pearson correlation coefficients with p<0.05.

## Results and discussion

### Determination of sample categories

Serum samples were categorized based on Total-C and Total-TG measurements using 230 mg/dL Total-C and 150 mg/dL Total-TG as cutoff values, chosen to achieve a more balanced distribution of the available samples among categories: normolipidemic (NL, N = 25, Total-C <230 mg/dL, and Total-TG <150 mg/dL); hypercholesterolemic (HC, N = 13, Total-C >230 mg/dL and Total-TG <150 mg/dL); hyperlipidemic (HL, N = 41, Total-C >230 mg/dL and Total-TG >150 mg/dL); and hypertriglyceridemic (HT, N = 31, Total-C<230 mg/dL and Total-TG>150 mg/dL). The measured mean, standard deviation and range of the analyte levels in the whole serum for each sample group are summarized in Table 1. For the purposes of this study, grouping the samples was useful because the differences between these sample categories aided the evaluation of possible biases in our analyte/Lp-P composition calculations. Furthermore, the lipid and protein composition of Lps in similar sample groups have been characterized in other literature reports using other fractionation and analytical techniques, providing a basis for comparison with our method.

**Table 1:** Summary of mean and standard deviation of analyte levels in whole serum for non-polar lipids, polar lipids and proteins by sample categories.

Analyte/Sample Category	NL (N = 25)		HC (N = 13)		HL (N = 41)		HT (N = 31)	
	Mean (Stdev)	Range	Mean (Stdev)	Range	Mean (Stdev)	Range	Mean (Stdev)	Range
*Non-polar lipids (mg/dL)*								
Total-C	181 (29)	124–217	269 (21)	232–311	280 (31)	232–355	188 (23)	135–226
Total-TG	74 (28)	33–136	90 (32)	50–144	279 (107)	155–573	268 (91)	161–650
Total-CE	140 (22)	97–167	218 (19)	180–255	225 (28)	182–294	147 (20)	106–198
Total-FC	41 (8)	27–56	52 (7)	35–62	56 (9)	31–89	41 (8)	20–58
*Polar lipids (μM)*								
Total-PC	2176 (404)	1273–2905	2539 (530)	1814–3897	2884 (671)	2016–5415	2219 (344)	1666–2912
Total-SM	443 (89)	316–629	653 (75)	549–745	601 (85)	412–879	442 (73)	339–602
Total-PE	162 (54)	64–275	183 (65)	91–291	341 (155)	136–866	269 (82)	134–472
Total-PI	58 (14)	34–91	65 (15)	43–92	90 (22)	55–168	68 (10)	51–92
Total-LPC	514 (82)	370–694	812 (232)	507–1362	878 (218)	508–1507	645 (188)	444–1557
*Apolipoproteins (μM)*								
Total apoA-I	48.8 (10.5)	35.5–75.4	57.7 (16.4)	33.4–92.9	51.7 (13.2)	33.8–91.7	43.9 (8.96)	25.7–70.3
Total-apoA-II	41.8 (8.6)	25.2–55.7	40.1 (10.1)	26.0–55.9	43.9 (11.5)	23.5–72.1	38.9 (6.9)	23.3–51.6
Total-apoA-IV	1.88 (1.01)	0.5–4.66	1.78 (0.59)	1.01–3.18	2.31 (0.98)	0.92–5.95	2.35 (0.87)	0.65–4.72
Total-apoB-100	1.28 (0.42)	0.79–2.5	1.91 (0.42)	1.14–2.71	2.47 (0.6)	1.35–3.75	1.69 (0.48)	1.06–3.13
Total-apoC-I	9.45 (3.48)	2.59–16.1	13.17 (3.52)	8.73–19.97	14.81 (6.14)	8.67–43.1	11.05 (2.05)	5.71–14.67
Total-apoC-II	2.92 (1.58)	1.16–7.86	4.47 (1.85)	2.61–8.71	7.22 (2.61)	2–17.05	5.43 (1.79)	1.98–9.99
Total-apoC-III	8.22 (4.33)	3.84–21.91	12.18 (5.7)	4.1–25.87	19.21 (6.73)	8.68–43.39	13.69 (4.41)	6.55–27.41
Total-apoE	1.29 (0.46)	0.64–2.15	2.06 (1.06)	0.80–4.57	2.30 (0.77)	1.13–3.97	1.96 (0.63)	1.04–3.87

Normolipidemic (NL, Total-C <230 mg/dL, and Total-TG <150 mg/dL), hypercholesterolemic (HC, Total-C >230 mg/dL and Total-TG <150 mg/dL), hyperlipidemic (HL, Total-C >230 mg/dL and Total-TG >150 mg/dL), and hypertriglyceridemic (HT, Total-C<230 mg/dL and Total-TG>150 mg/dL) sample categories.

### Evaluation of size resolution

**Fig 3:**
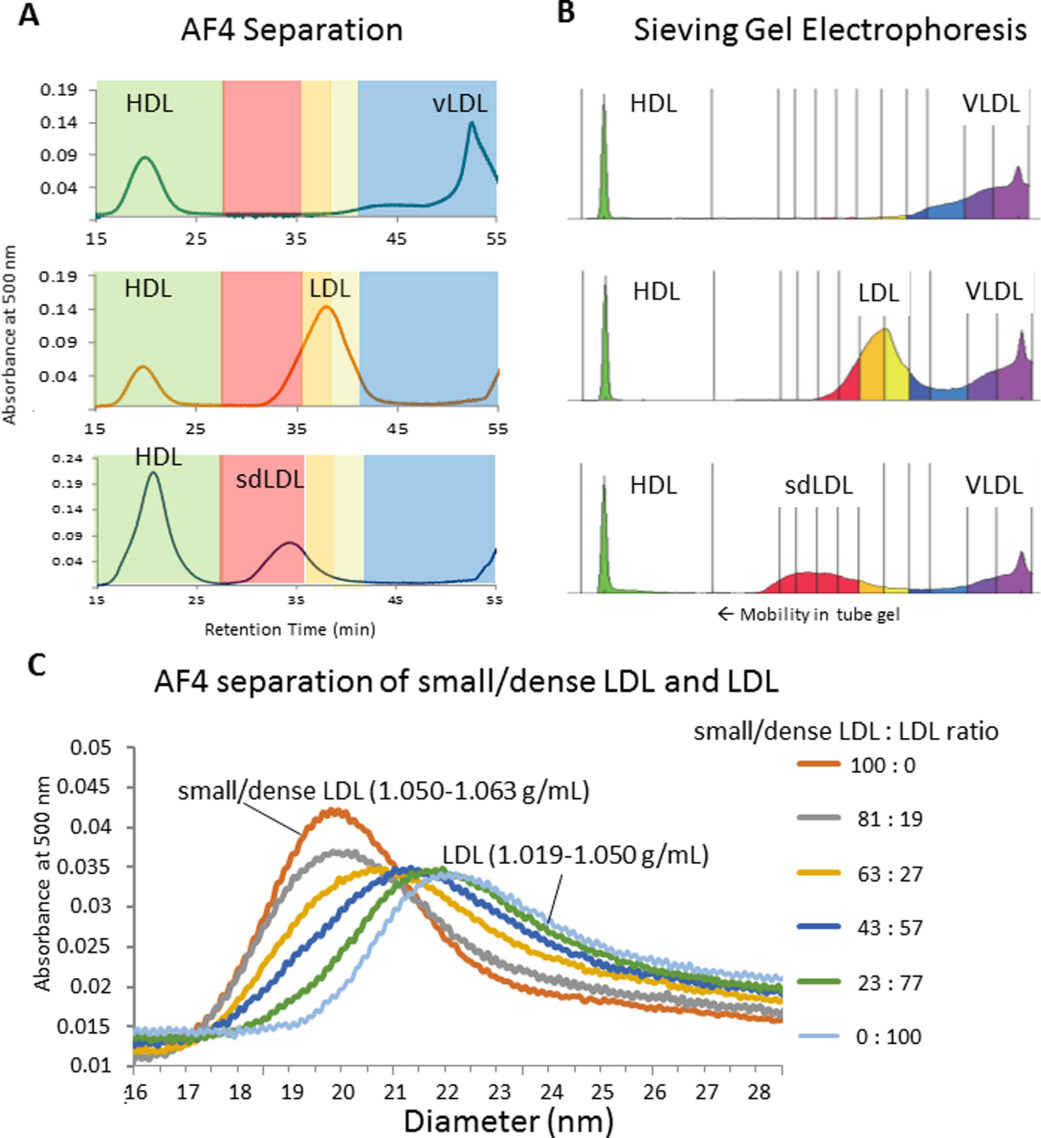
Fractograms of various mixtures of lipoproteins prepared from mixtures of density fractions. HDL (1.063–1.21 g/mL), small/dense LDL (1.050–1.063 g/mL), LDL (1.019–1.050 g/mL) and vLDL (<1.019 g/mL). A: HDL + VLDL (top), HDL + LDL (middle) and HDL + small/dense-LDL (bottom), detection by continuous post-infusion of cholesterol reagent after the AF4 channel and detection at 500 nm wavelength. B: Sieving gel electrophoresis separation of the same mixtures with sudan black staining. C: Overlay of AF4 fractograms from various mixtures of small/dense LDL (1.050–1.063 g/mL) and LDL (1.019–1.050 g/mL).

Volumetric particle number calculation requires the knowledge of particle size necessitating fractionation by size. Density is not an applicable physical characteristic for this purpose. However, to confirm the approximate correspondence between Lp fractions obtained by AF4 and the traditional classification system of Lps, which is based on density, we performed AF4 fractionation of density purified HDL (1.063–1.21 g/mL), small/dense LDL (1.050–1.063 g/mL), LDL (1.019–1.050 g/mL) and vLDL (<1.019 g/mL). We injected mixtures of HDL+VLDL, HDL+LDL, and HDL+small/dense-LDL into the AF4 system while monitoring the separation of Lps with post infusion of a colorimetric cholesterol reagent and UV/VIS detection at 500 nm wavelength following a published procedure [20] (Fig 3A). The experiments confirmed the comparability of the Lp classes fractionated by AF4 and ultracentrifugation. The same mixtures were also analyzed using a commercial sieving gel electrophoresis system that is clinically approved for size fractionation of LDL subclasses (Lipoprint, Quantimetrix, CA, USA) (Fig 3B), showing that the resolution of LDL sub-fractions by the two size fractionation techniques is comparable. We also combined the small/dense-LDL and LDL mixtures in various ratios while we observed the gradual shift in AF4 retention times between 19.5 and 22 nm hydrodynamic size (Fig 3C). Of note, the size resolution between small and large LDL in these experiments appears somewhat less relative to the actual size resolution in the AF4 channel. This was due to band broadening in the cholesterol reactor tube because upon mixing with the cholesterol reagent, Lp particles become diffusive small molecules.

**Fig 4:**
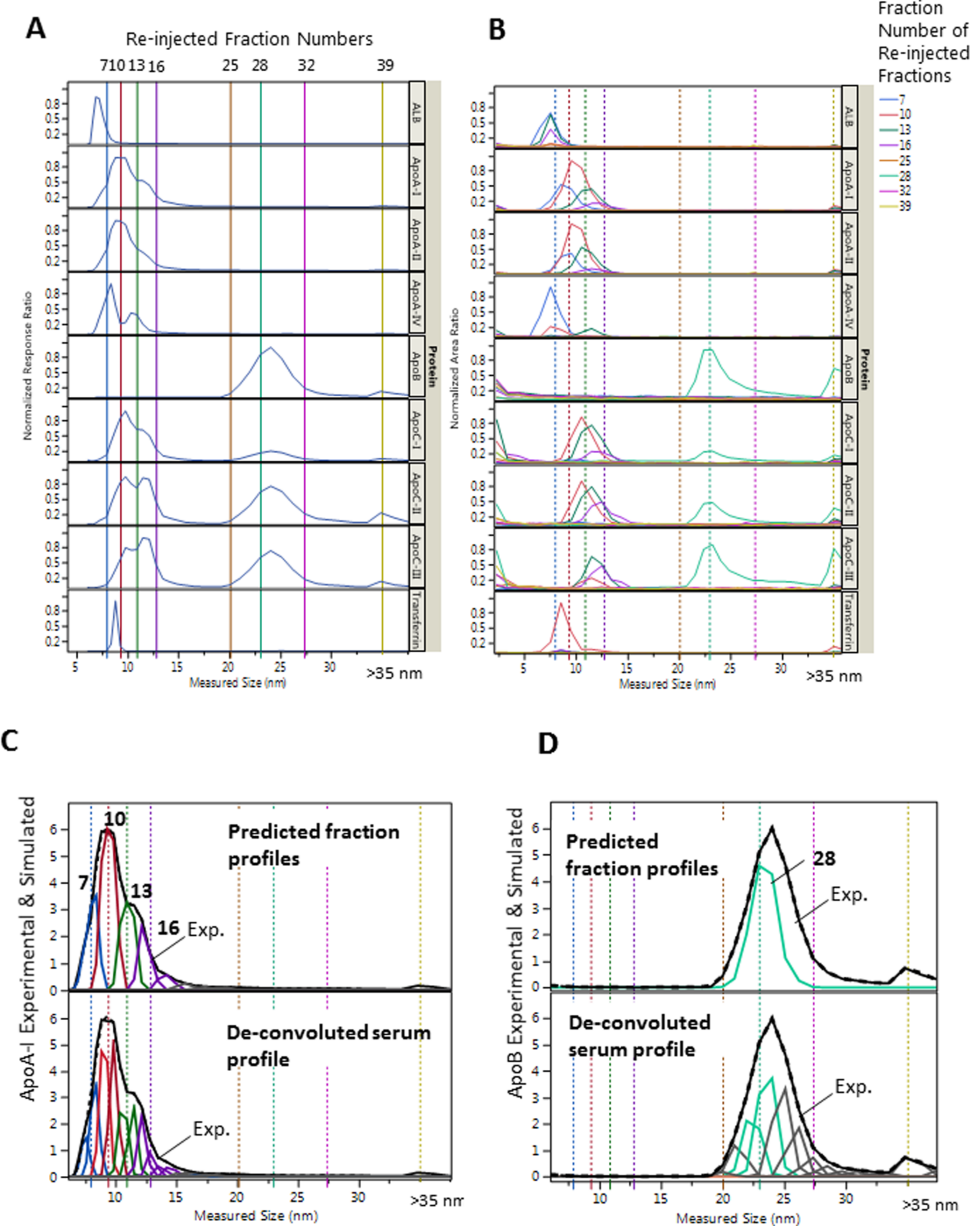
Size profiles from serum and corresponding fractions. **A**: Size profile of representative proteins from serum (50 μL injection volume). **B**: Size profile from re-injected AF4 fractions indicated by colors (200 μL injection volume). **C**: De-convolution of the apoA-I profile in A (bottom) and predicted fraction profiles (top). **D**: De-convolution of the apoB profile in A (bottom) and predicted fraction profiles (top), generated by summing together de-convoluted Gaussian peaks. Re-injected fractions are indicated by numbers on the top and by the colors of solid vertical lines in **A**, matched with the color of the overlaid size profiles in **B**. The dotted vertical lines in **B** correspond with lines in **A**. The colors of the predicted profiles in **C** and **D** matched with the colors of the size profiles of the fractions for apoA-I and apoB in **B**.

To demonstrate that AF4 separates HDL and LDL sub-classes by size, we selected AF4 fractions of a serum pool and reinjected/analyzed them for Lp size distribution of protein constituents. The overlay of size profiles obtained from the re-injection the AF4 fractions ±3 fractions away from each other showed significant differences (Fig 4). This difference between re-injected profiles was observed in spite of the somewhat greater band broadening due to the larger injection volume (200 μL for fractions), relative to the injection volume of the whole serum sample (50 μL). The larger injection volume was necessary because of the 10–20 fold lower particle concentration in the fractions of the re-injected fractions. The apoCs were present along with apoB during the collection and re-injection of the main apoB containing (fraction 28). However, we observed the increased presence of apoCs in the first and last fractions (<6 nm and >35 nm), as well as increased tailing in the LDL size range of the re-injected fractions; indicating the dissociation of apoCs from LpB particles and aggregation which seemed to be dependent on the time elapsed between the collection and the re-injection of the fractions.

**Fig 5:**
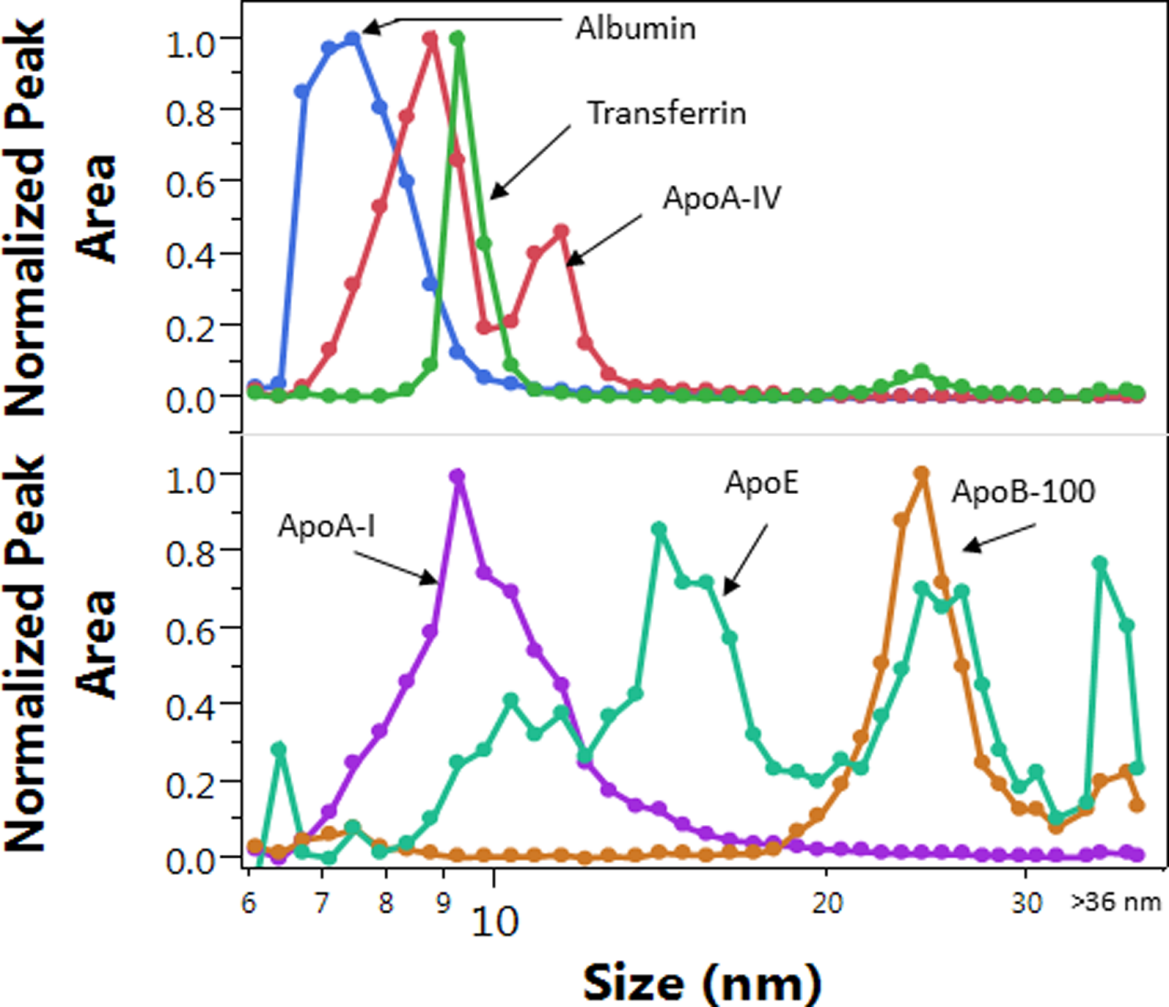
Overlay of size distribution profiles of selected proteins from a single sample. LC-MS/MS peak areas were normalized by the maximum peak area for overlay.

The efficacy of size resolution during routine analysis is best evaluated based on the size profile of proteins from the injection of 50 μL serum (Fig 5). Transferrin had the least size dispersion of all proteins and showed ~1 nm and ~2 nm peak-width at half-peak height and base line, respectively. The accuracy of the size measurements and the conversion of the fraction numbers to hydrodynamic size (described in the Methods section) was verified based on the size profile maxima of human serum albumin at 7.2±0.5 nm, and transferrin at 9.1±0.5 nm (Fig 5); 0.5–1 nm bias relative to literature reported values [39–41]. The reproducibility of the size measurements in the upper size range was evaluated based on the size measured at the apoB-100 size profile maximum of the AF4 QC pool, 23±0.6 nm (N = 25).

**Fig 6:**
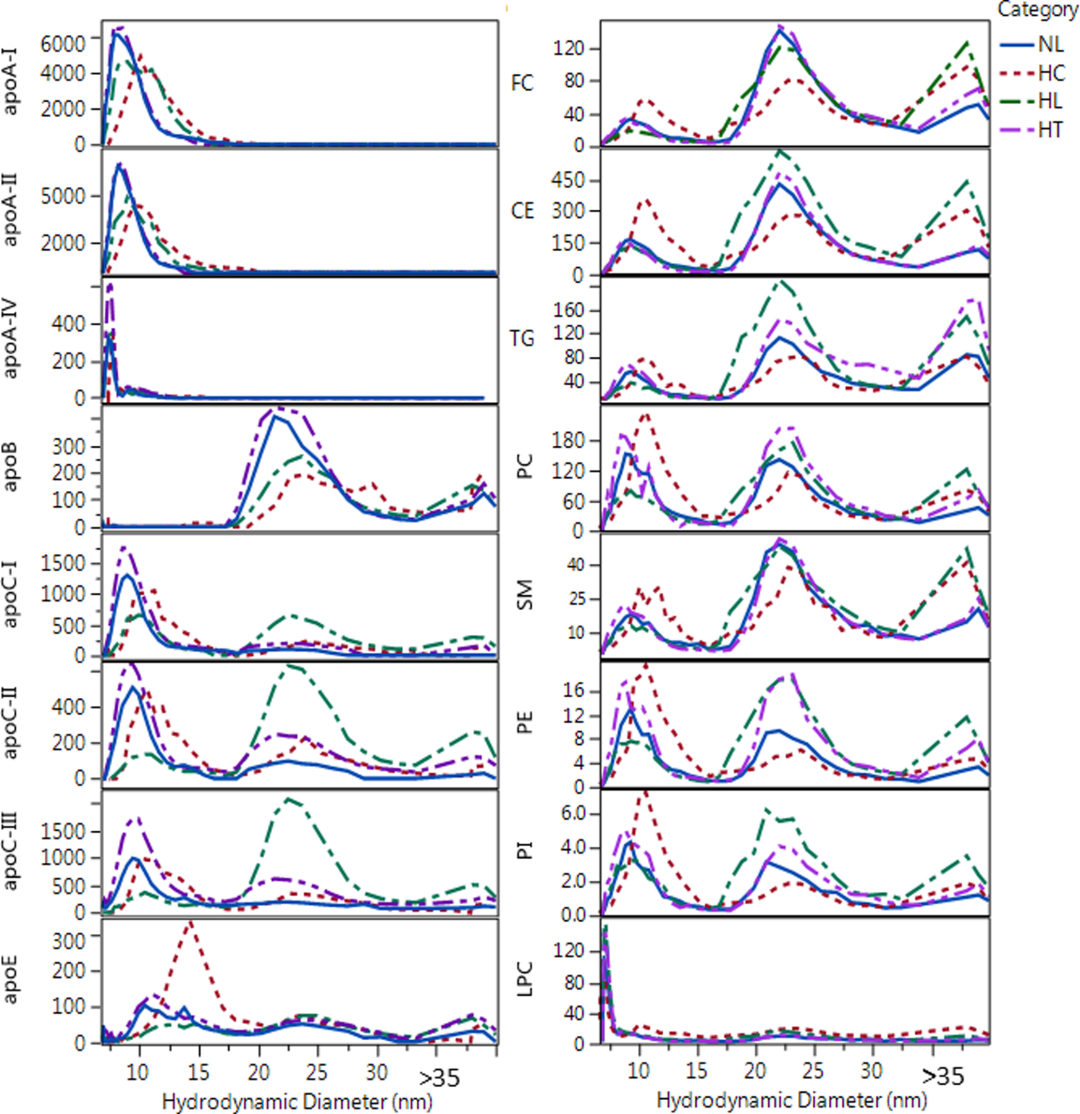
Overlay of size distribution profiles of apolipoproteins and lipids for representative samples from different categories. Labels indicate normolipidemic (NL, Total-C = 206 mg/dL and Total-TG = 130 mg/dL), hypercholesterolemic (HC, Total-C = 249 mg/dL and Total-TG = 121 mg/dL); hyperlipidemic (HL, Total-C = 287 mg/dL and Total-TG = 180 mg/dL); and hypertriglyceridemic (HT, Total-C = 212 mg/dL and Total-TG = 188 mg/dL). Y axis for apolipoprotein concentrations are in nM and for lipids are in μM units.

Additional evidence of size resolution was the size profile of apoA-IV. The ApoA-IV size profile in most samples had two peaks with ~2 nm size difference (Fig 5). The two populations of apoA-IV containing particles had ~8 nm and 9–11 nm size, the latter of which were substantially larger than the size determined by gradient gel electrophoresis [42]. This may be due to the gentle nature of the AF4 technique (no forces from interaction with the pores of any packing material), and better preservation of the lipid-bound forms of apoA-IV during separation. Significant differences were found in the maxima and shape of the size distribution profiles of the different Lp constituents both within and between individual subjects (Fig 6). These differences are further evidence for the resolution of numerous HDL and LDL subspecies in serum by AF4.

Several proteins were only monitored but not quantified (examples are shown in S5 Fig). The size ranges where these proteins appeared were typically much larger than expected based on their molecular weight. Therefore, these proteins had to bind to particles much larger than their own size; but the size itself and overlap with the apoA-I profile cannot provide clear evidence that these particles are HDL particles. Interestingly, proteins such as apoL1 and apo(a) are usually reported to be in the higher density HDL and LDL fractions, respectively. These higher density Lps are often assumed to also be smaller in size. On the contrary, we found these proteins in the large HDL and VLDL size range, respectively. This is further evidence that size and density are not interconvertible properties of Lp sub-fractions. Small non-apoA-I containing particles which carry lipids (e.g albumin) can be low density. In reverse, large particles which contain more proteins and less lipids may have high density.

AF4 separation is based on hydrodynamic size only (i.e. diffusion coefficient in free solution). In theory, individual HDL and LDL sub-species are expected to migrate independently inside the AF4 channel and the band broadening is independent of particle size. Therefore, all Lp particles of similar size are expected to elute from the AF4 channel with similar band broadening and resolution as transferrin and apoA-IV. This is in contrast to size exclusion chromatography where phase transfer delay (between free solution and pores) inherently causes gradually increasing band broadening with increasing retention time. Therefore, even with some increase in band broadening in the upper size range (due to some interaction with the membrane), the AF4 technique allows near base-line separation of at least 5 HDL particle sub-populations in the 7–18 nm range, and 3 LDL particle populations in the 18–30 nm range (Fig 5). The various size profiles of the monitored proteins indicate the presence of numerous particles with wide range of size and composition which causes the actual apoA-I and ApoB-100 size profiles to appear as one continuum. The preservation of the peak width of individual components, and taking full advantage of the ~2 nm base line resolution as represented by transferrin, required collection of 3–4 fractions across 2–3 nm size increments. This typically meant the collection of 3 fractions with < 7 nm containing mainly albumin (ALB), 12–13 fractions within 7–13 nm (HDL), 8–9 fractions within 13–18 nm containing HDL and apoE containing particles (LpE), 10–13 fractions within 18–30 nm (LDL), and an additional 2–5 fractions within 30–36 nm (IDL) and 2–3 purge fractions (VLDL).

For this study, we optimized the AF4 system mainly for the separation of HDL and LDL subclasses while keeping the total run time at 120 min, including 90 min gradient cross flow time and 30 min purge with the cross flow turned off. In order to achieve 120 min run time and be able to collect concentrated fractions for MS analysis, we had to use AF4 in gradient cross flow mode. Without gradient cross flow each run would have taken over 3 hours with significantly larger elution volume and several fold greater dilution in the >30 nm size range. Because of the time constraints and the required minimum fraction concentration for LC-MS/MS analysis (0.1 nmole/L for proteins), remaining IDL/VLDL particles were purged out of the AF4 channel into the last 2–3 fractions. Furthermore, because of batch-to-batch variations in retention times (shifts by 1–3 fractions), the number of fractions that could be collected >30 nm also varied, and the particle size could not be measured as accurately. Without accurate size information, the particle size and analyte/Lp-P in the fractions >30 nm could be interpreted only qualitatively or in terms of analyte/analyte molar ratios (independent of particle number).

The effect of sample storage conditions on size resolution and total HDL and LDL protein concentrations were examined by analysis of aliquots from the same AF4-QC; fresh vs. stored at 4°C and -80°C for 24 hours, and after the first thaw during 8 month storage at -80°C. Both the size profile differences and the change over time in the sum of concentrations in the HDL or LDL fractions remained in the range of inter-day method variability (S6 Fig).

### Calculation of AF4 fractionation recoveries

The AF4 recoveries were calculated by analyte for each sample. This was necessary because quality control was set up by analyte. If an analyte did not pass quality control, the run was repeated either on the respective instrument or through the entire AF4-LC-MS/MS workflow for the failed analyte. For samples with <200 mg/dL Total-TG, the average recovery was 52% (Std Dev 15%) for apoA-I, 46% (Std Dev 17%) for apoB, 72% (Std Dev 12%) for PC, 66% (Std Dev 14%) for TC (CE+FC), and 33% (Std Dev 17%) for Total-TG (S7 Fig). Within each AF4-LC-MS/MS run, the AF4 recoveries showed significant (p<0.01) correlation between analytes with correlation factors in the range of 0.7–0.9. Thus, when we calculated analyte/Lp-P and analyte/analyte molar ratios, the effect of the AF4 recoveries, to a large extent, cancelled out.

Exceptions were apoE and TG which showed significant but low (0.3–0.4 and 0.5–0.6, respectively) recovery correlations with other analytes. For apoE, this was due to higher, 25% CV of the LC-MS/MS analysis and low concentration relative to other proteins, and for TG due to large size TG rich particles in the samples. Some very large or aggregated particles were likely lost during the AF4 injection/focusing. Some very high Total-TG samples, >400 mg/dL were visibly cloudy. The formation of these visibly large particles may have occurred in vivo (lipemic samples) or during sample freezing, storage and thawing. All serum samples were vortexed before the sample aliquots were taken, which itself caused the Total-TG, Total-C and Total-PL measurements to be ~20% higher vs. after leaving the sample to settle in the vials. The low TG recovery in the AF4 fractions due to the loss of the large or aggregated particles meant that by substitution of the AF4 recovery into Eq 3, the TG fraction concentrations potentially became overcorrected for the samples with high Total-TG levels. This potential bias had to be considered during the interpretation of differences between sample categories (discussed below).

### Number of apoA-I and apoB-100 molecules per particle

Calculated analyte/Lp-P values (number of analyte molecules per one particle) were affected by several assumptions: 1) 1 hydration layer with 0.3 nm thickness was assumed around the particles which was subtracted from the measured hydrodynamic size to calculate dehydrated particle diameter (Eq 9); 2) all lipoproteins were assumed to have a spherical particle shape (vs. ellipsoid or discoidal); 3) the partial molecular volumes used for the total surface and core volume calculations were assumed to reflect the average molecular volume within lipid classes (PL, CE and TG) regardless of composition of individual species with various acyl carbon chain length and number of double bonds; 4) all proteins were assumed to have the same partial specific volume (1.212 nm^3^/kDa); 5) particles were assumed to contain no other proteins but those we measured; 6) assumed no significant loss of lipids due to lipase enzyme activity during the AF4 separation and between fraction collection and distribution of aliquots for LC-MS/MS analysis; 7) we assumed that all particles injected into the AF4 system were eluted into the fractions, and in the case of particle loss all analytes were effected equally.

From the above assumptions, in terms of potential bias, the accuracy of the average dehydrated diameter measurement in the fractions is expected to have the strongest effect on the calculated Lp-P and analyte/Lp-P values. As can be seen from Eq 12, the calculated analyte/Lp-P values are proportionate with the third power of the dehydrated diameter, (d_DLS_-2*n*w_h_)^3^. As we showed with the re-injection of the AF4 fractions (Fig 4), each fraction also contained particles from adjacent fractions ±2 fractions away due to ±1–2 nm band broadening of monodisperse particle populations, which also depended on their relative concentration. The average size at the apoB size profile maxima was 22–23 nm in NL samples. We believe that this size, relative to the range of literature reported values, is at the lower end, therefore, under-estimated rather than over-estimated. This means that our size measurements by DLS are not a significant source of positive bias in the calculated analyte/Lp-P values.

The assumption of spherical vs. ellipsoid (i.e. discoidal) particle shape also has a potentially strong effect on the Lp-P calculations. The volume of discoidal or ellipsoid shaped particles can be estimated by ¾*π*d_a_*d_b_*d_c_, where d_a_<d_DLS_ and d_b_ = d_c_ = d_DLS_. Reducing d_a_ yields a proportionate decrease in analyte/Lp-P values in Eq 12.

The bias from the possible inaccuracy of the partial molecular volumes of PLs, CEs, TGs and proteins can be magnified by the concentration of these constituents in the fractions, because the core and surface volumes are the sum of [concentration]*[partial molecular volume]. In Eq 12, the sum of the core and surface volumes is in the denominator. This means that positive or negative biases in the partial molecular volumes (or analyte concentrations) affect the calculated analyte/Lp-P in a reciprocal manner. In the case of HDL particles, the contribution of the total protein volume affects the total surface volume the most. In the case of LDL particles, the TG molecular volumes affects the analyte/Lp-P values the most, especially in the serum samples with high Total-TG levels.

Although the above potential biases may indeed exist, nevertheless they consistently affect all calculated Lp-P and analyte/Lp-P values along the particle size scale and across sample categories. Furthermore, each analyte concentration and average hydrodynamic size (d_DLS_) in the fractions were independently measured variables, and each constituent contributed only partially to the core and surface volumes in Eqs 5 and 6. Therefore, the calculated analyte/Lp-P still reflects meaningful average compositional differences along the particle size scale and across sample categories. As a proof for the accuracy of our Lp-P and analyte/Lp-P calculations, first we looked at the number of apoA-I/Lp-P and apoB-100/Lp-P and compared them with literature reported values.

#### Number of apoA-I/particle

**Fig 7:**
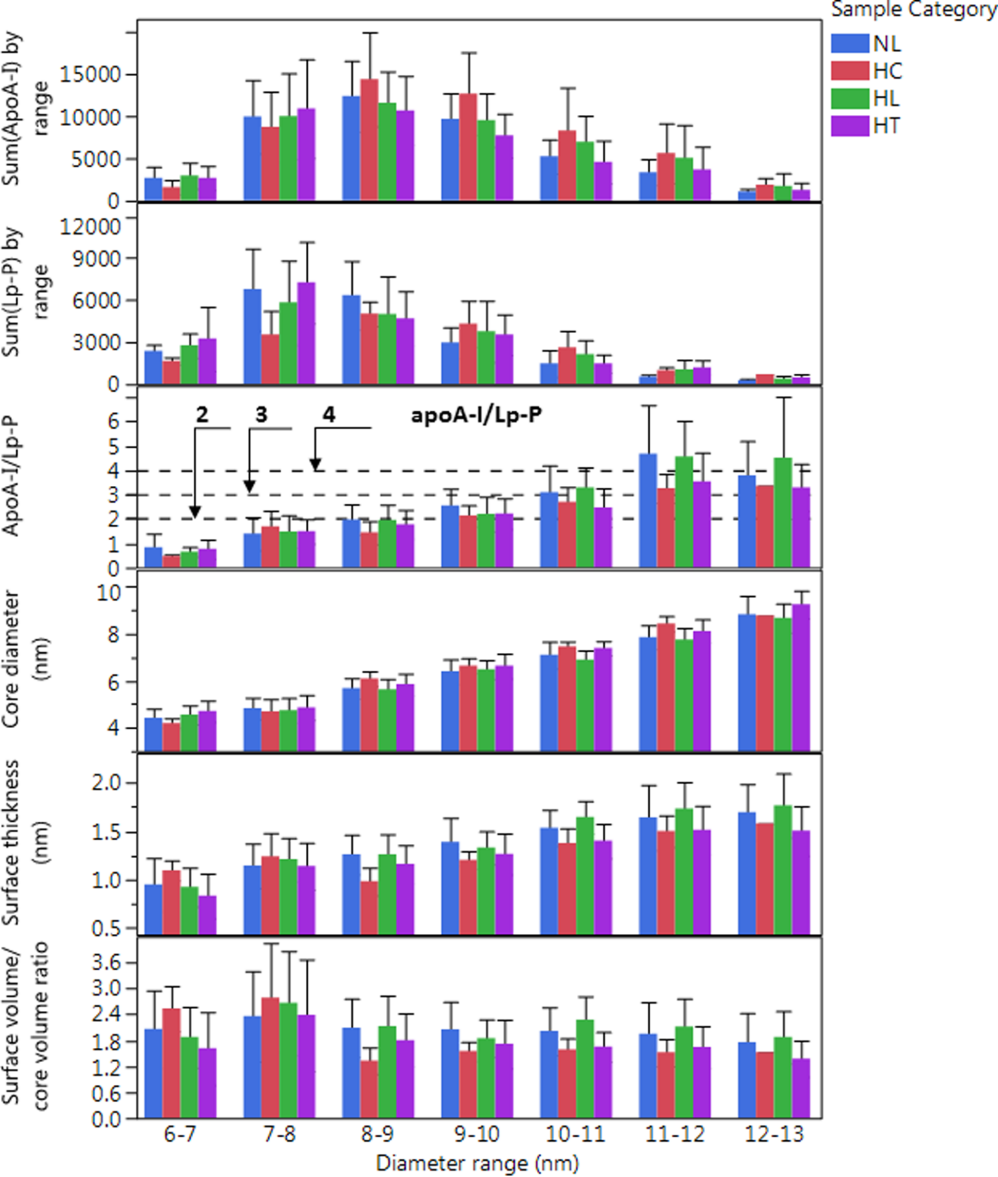
Sum of ApoA-I, sum of calculated Lp-P, average ApoA-I/Lp-P and other average HDL particle characteristics by diameter range in different sample categories. Labels indicate normolipidemic (NL, N = 25, Total-C <230 mg/dL, and Total-TG <150 mg/dL); hypercholesterolemic (HC, N = 13, Total-C >230 mg/dL and Total-TG <150 mg/dL); hyperlipidemic (HL, N = 41, Total-C >230 mg/dL and Total-TG >150 mg/dL); and hypertriglyceridemic (HT, N = 31, Total-C<230 mg/dL and Total-TG>150 mg/dL). Error bars indicate standard deviation.

Studies using the combination of apoA-I immuno-capture and cross-linking [43–45] or the volumetric approach [26] showed LpA particles to contain 2–4 apoA-I/Lp-P. In the 8–12 nm range, at the HDL-P maxima, apoA-I/Lp-P values were in the expected range of 2–4 (Fig 7). We also found mean A-I/A-II mole/mole ratios in the range of 1–1.5, in agreement with previous studies [26, 30] where the most abundant HDL particles were found to contain 3–4 apoA-I or 2–3 apoA-I with 2 apoA-II. As the individual size profiles of the proteins shows in Fig 6, some of the HDL particles also contained other proteins, including apos C-I, C-II, C-III and E.

In the 7–8 nm size range 1–1.5 apoA-I/Lp-P was found assuming a spherical shape, lower than the expected 2 apoA-I/Lp-P on small HDL particles that are generally assumed to have a discoidal shape. However, calculation with discoidal instead of spherical particle shape would have lowered further the calculated ApoA-I/particle values. Therefore, assumption of spherical particle (vs. discoidal) shape is a source of positive bias and rather masks another more significant negative bias. The reason for the negative bias can be the calculation with more surface and core molecular volume (denominator in Eq 12) relative to the moles of apoA-I (numerator in Eq 12). This can be the result of higher concentration of lipids due to co-elution of lipidated non-LpA particles; such as containing albumin, apoA-II, apoA-IV and possibly others (i.e. clusterin and serum amyloid A). On the contrary, in the 13–15 nm large HDL size range, the apoA-I/Lp-P values may have a positive bias because apoA-I may reside on particles that contain less lipids but more proteins that were not accounted into the surface volume (underestimation of the denominator in Eq 12). In case of larger HDL particles the protein volume may be also underestimated by not accounting for a less folded and more hydrated apoA-I structure on the surface.

#### Number of apoB/particle

**Fig 8:**
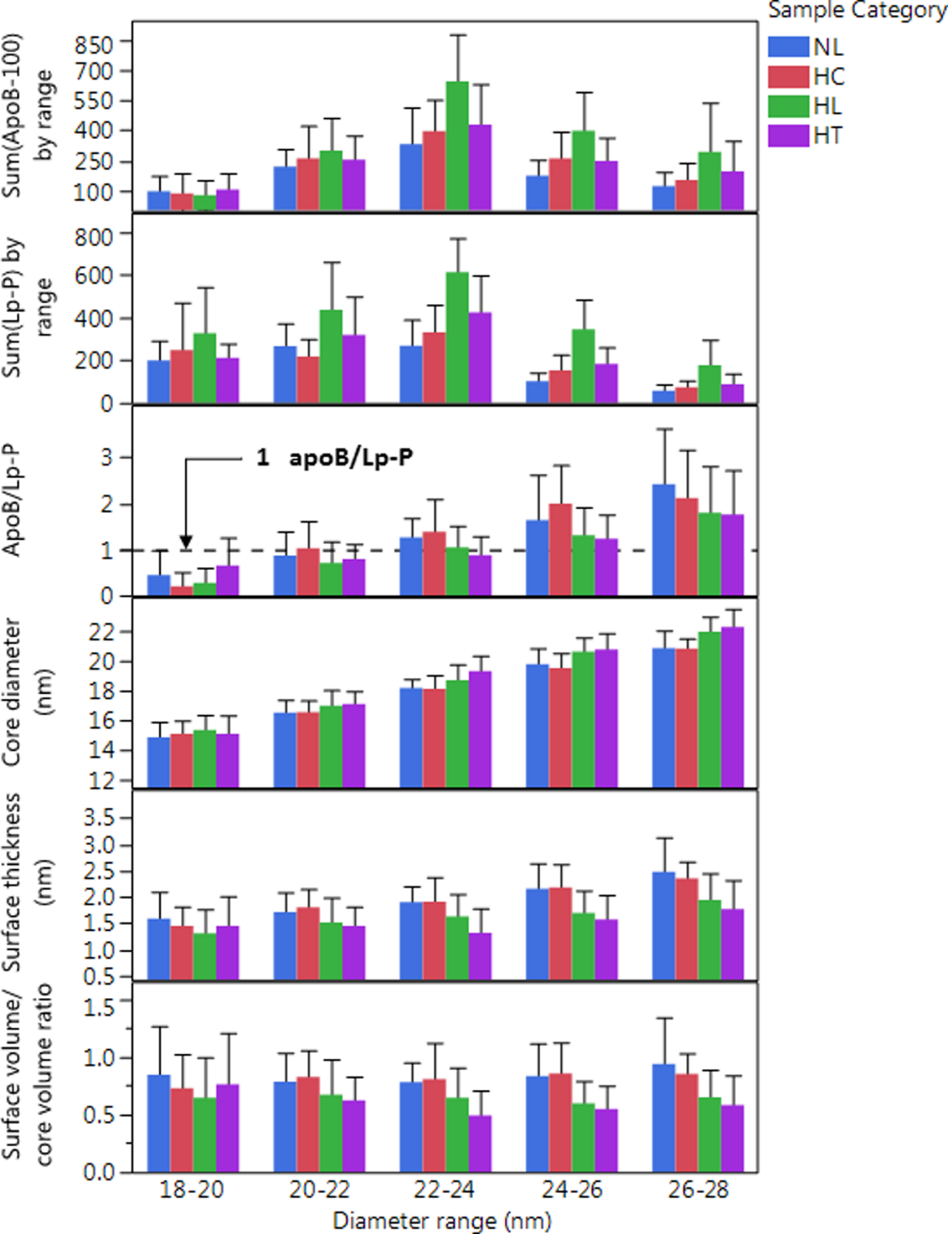
Sum of ApoB-100 and sum of Lp-P, average calculated apoB100/Lp-P and other average LDL particle characteristics by diameter range in different sample categories. Labels indicate normolipidemic (NL, N = 25, Total-C <230 mg/dL, and Total-TG <150 mg/dL); hypercholesterolemic (HC, N = 13, Total-C >230 mg/dL and Total-TG <150 mg/dL); hyperlipidemic (HL, N = 41, Total-C >230 mg/dL and Total-TG >150 mg/dL); and hypertriglyceridemic (HT, N = 31, Total-C<230 mg/dL and Total-TG>150 mg/dL). Error bars indicate standard deviation.

Near the apoB-100 profile maxima, 20–24 nm, assuming spherical particle shape, our calculations yielded 0.8–1.2 apoB-100/Lp-P (Fig 8), closely matching the expected one apoB-100 molecule per LpB particle stoichiometry. At the smaller LDL particle size range, 18–20 nm, the apoB-100/Lp-P was significantly less than one (0.5–0.8), which was possibly due to some co-eluting lipid particles without apoB-100, most likely lipidated apoE containing particles.

In the larger LpB particle size range, 26–30 nm, the average apoB-100/Lp-P was found to be 1.5–2.5. Similar positive bias with increasing size relative to the expected apoB-100/Lp-P ratio of 1 was also observed by other studies using the volumetric approach [27, 28]. McNamara et al. concluded that on larger vs. smaller size LDL particles apoB-100 may occupy a larger surface volume, i.e. being “thicker” [27]. Indeed, according to Eq 12, if the total molecular volume in the denominator is larger, the calculated apoB/Lp-P would be smaller. An equivalent of the underestimation of the protein volume is the underestimation of the number and thickness of the hydration layers around the particles (n and w_h_ in Eqs 9 and 12) which is subtracted from the measured hydrodynamic diameter (d_DLS_).

Another possible explanation for the higher than expected apoB-100/Lp-P values is the overestimation of the dehydrated particle volume because of the assumption of spherical particle shape. This explanation was given by Teerlink et al who concluded that larger LDL particles may have a discoidal shape [28]. Discoidal shaped LDL particles were also directly observed using cryo-electron microscopy [46, 47]. The discoidal particle volume can be estimated with the volume of an ellipsoid (¾*π*d_a_*d_b_*d_c_, where d_a_<d_DLS_ d_b_ = d_c_ = d_DLS_) in Eq 12. If d_a_ is ½ or ¾ of d_DLS_ the dehydrated particle volume and the calculated apoB-100/Lp-P values would be proportionately less and closer to 1. However, in the large LDL or IDL size range, ~30 nm, a significant positive bias would still remain.

Considering the high mole% of lipids in LDL particles, the source of the positive bias can also be the underestimation of the partial molecular volume of PL, CE and TG molecules. This explanation is consistent with the apparent gradual increase of apoB/Lp-P with size as seen in Fig 8; a trend that continues in the >30 nm size region, where even with consideration that the apoB concentration in these fractions and the particle size was likely underestimated, the found 4–6 apoB/Lp-P is still significantly high (S8 Fig).

The effect of the AF4 recoveries on the apoB/Lp-P calculations also needs consideration. The overcorrection of the TG concentrations in the fractions (higher denominator in Eq 12) explains that we saw the lowest apoB/Lp-P values for HT samples of the four analyte groups throughout the LDL size range (Fig 8). Therefore, the comparison of the calculated apoB/Lp-P in the different sample groups reveal that the overestimation of the TG concentration did affect our calculations, but also showed that this overestimation is masking other biases that leads to higher than expected apoB/Lp-P values in the large LDL size range. According to our calculations with incorporation of correction factors, the expectation of one apoB/Lp-P in the NL and the HC sample group can only be accomplished with a ~30% increase of all the PL, CE and TG partial molecular volumes, in addition to the also possible discoidal shape (d_a_ = 0.8*d_DLS_) and more apoB hydration with ~30% increase of the partial specific volume.

### Estimated core diameter and surface thickness of HDL and LDL particles

We observed that LDL particles with higher TG/CE ratio (HL and HT samples) yielded significantly larger calculated core diameter, smaller surface thickness and smaller surface/core volume ratio (Fig 8). These observations can be explained partly by the overestimation of the TG concentrations in the fractions for samples with very high Total-TG levels; due to overcorrection of the TG concentrations in the AF4 fractions (discussed above). Incorporation of a correction factor into the calculations of the surface/core volume ratios revealed that at 22–24 nm only ~15% reduction of the TG concentrations in the HL/HT fractions would be necessary to diminish the difference between NL/HC and HL/HT samples, without correcting for other possible biases. However, in the 26–28 nm size range a much greater, ~60% reduction of the TG concentrations would be needed, suggesting that other biases may affect our calculations as well. We can only speculate about the source of these other biases, such as assumptions of average molecular volumes without consideration for carbon chain lengths and saturation of acyl groups, molar ratios FC/PL, SM/PL and PE/PL within the surface, and distribution of FC and TG molecules between the surface and the core of Lp particles. Indeed, samples from NL and HC subjects showed increasing FC/PL and SM/PL ratios as a function of size of both HDL and LDL particles, and PE/PL and SM/PL ratios were significantly different in NL/HC vs. HL/HT samples (discussed below).

In summary of our apoA-I/Lp-P and apoB/Lp-P calculations, it is important to emphasize that in the main apoA-I and apoB containing fractions, on average, we did find apoA-I/Lp-P and apoB/Lp-P values that closely matched the reports from previous studies. In the minor small and large HDL and LDL fractions, discrepancies were also found by other studies, and can be explained by likely biases that originate from the assumptions used in our calculations.

### Number of exchangeable apolipoprotein and lipid molecules per particle as function of particle size

**Fig 9:**
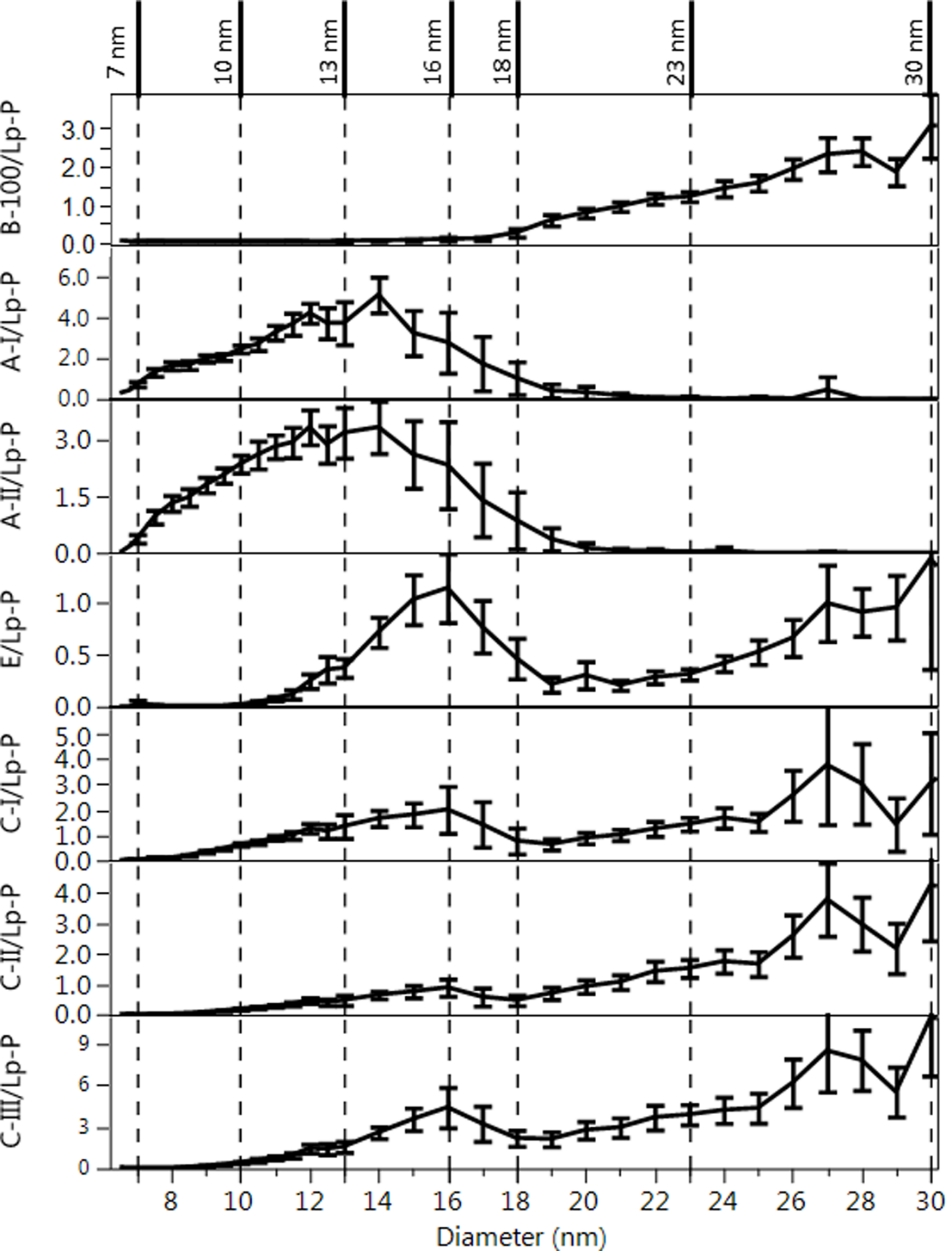
Average number of apo/Lp-P vs. size profiles. Error bars indicate 95% confidence intervals based on 110 serum samples. Vertical dashed lines indicated the borders of size ranges with significantly different composition as discussed in the text. Corresponding size ranges are indicated in Fig 10.

**Fig 10:**
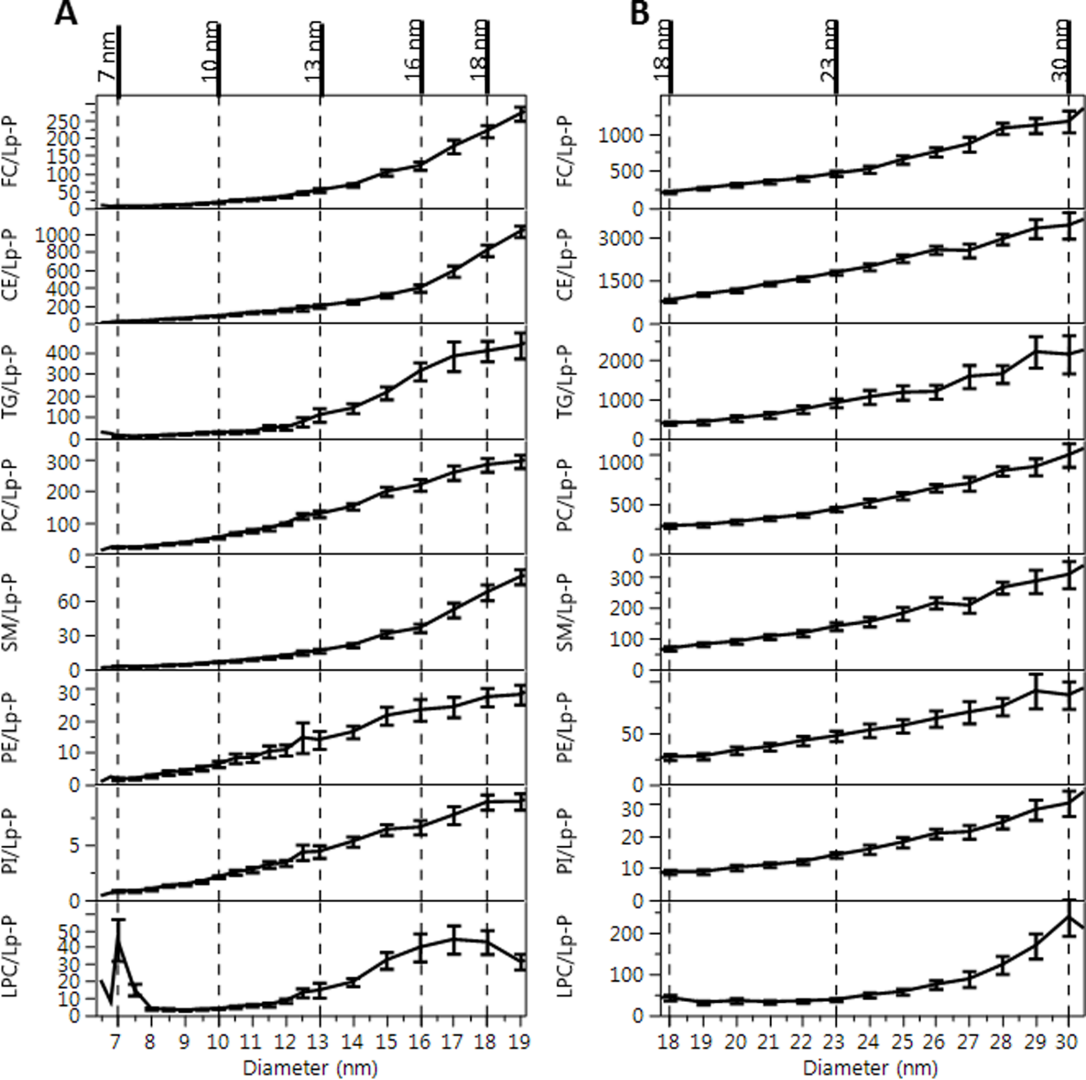
Average lipid/Lp-P vs. size profiles. A: HDL particle size range, B: LDL particle size range. Error bars indicate 95% confidence intervals based on 110 serum samples. Vertical dashed lines indicated the borders of size ranges with significantly different composition as discussed in the text. Corresponding size ranges are indicated in Fig 9.

For characterization of the composition of Lps as a function of size, analyte/Lp-P molar ratio profiles were calculated as shown in Figs 9 and 10. Analogous analyte/Lp-P profiles by NL, HC, HL and HT sample categories are shown in S9 Fig. In all these figures, Lp-P was calculated with the assumption of the same partial molecular volumes and spherical particle shape across the different size ranges and sample categories. Therefore, in terms of relative trends and differences, these analyte/Lp-P profiles reveal information about the relative composition of HDL and LDL sub-fractions.

In fractions <7 nm, a significant amount of LPC was measured in the corresponding presence of albumin but with lack of apoA-I (Fig 10), suggesting albumin as an important carrier of LPC.

Between 7–13 nm, the HDL size range can be further divided into 7–10 and 10–13 nm ranges based on significant composition differences. In the 7–10 nm size range apoE/Lp-P and apoCs/Lp-P remained <0.5, while apoA-I/Lp-P and apoA-II/Lp-P increased 0.5–2.5 (Fig 9). In the 10–13 nm size range, apoA-I, apoA-II, apoE, apoC-I, apoC-II and apoC-III per Lp-P increased to around 4.5, 3.5, 0.5, 2.0, 1.0, and 2.0, respectively. Throughout the 7–13 nm range, FC/Lp-P, PC/Lp-P and SM/Lp-P increased proportionately with particle surface area (d_DLS_^2^) (Fig 10). The total number of PL molecules per Lp-P in the 7–10 nm and 10–13 nm range were 60–95 and 155–175, respectively, comparable to the number of PC molecules typically used in HDL modeling studies [6] and to the generally reported 150 PL molecules per HDL particle [3]. The size and composition of the 7–10 nm and 10–13 nm size fractions seems to be the equivalent of the main HDL sub-fractions found in previous studies, HDL3 and HDL2 by ultracentrifugation or α3/4 and α2 HDL by gradient gel electrophoresis, respectively [48].

Between 13–18 nm, the calculated lipids/Lp-P increased in proportion with the expansion of particle volume and surface area (Fig 10) while apoA-I/Lp-P and apoA-II/Lp-P did not. Between 13–16 nm, with increasing size a decrease of apoA-I/Lp-P from 4.5 to 2 and apoA-II/Lp-P from 3.5 to 2 were found. On the contrary, the decrease of apoA-I/Lp-P and apoA-II/Lp-P corresponded with the increase of apoE/Lp-P (0.5 to 1), apoC-I/Lp-P (2 to 3), apoC-II/Lp-P (1 to 2) and apoC-III/Lp-P (2 to 4) (Fig 9). From 16–18 nm, the number of all apos per Lp-P decreased in sharp contrast with the number of lipid constituents per Lp-P, which increased or remained at similar values with increasing size. At 18 nm apoB-100 was also detectable. The above analyte/Lp-P trends in the 13–18 nm range are consistent with previous studies that reported large LpA particles (also called α1-HDL), apoE containing particles without apoA-I, and small LpB particles [48–50]. The relatively high UV absorbance signal recorded during the AF4 separation in this size range suggests the presence of numerous other proteins besides the apos measured by our LC-MS/MS method (Fig 1).

**Fig 11:**
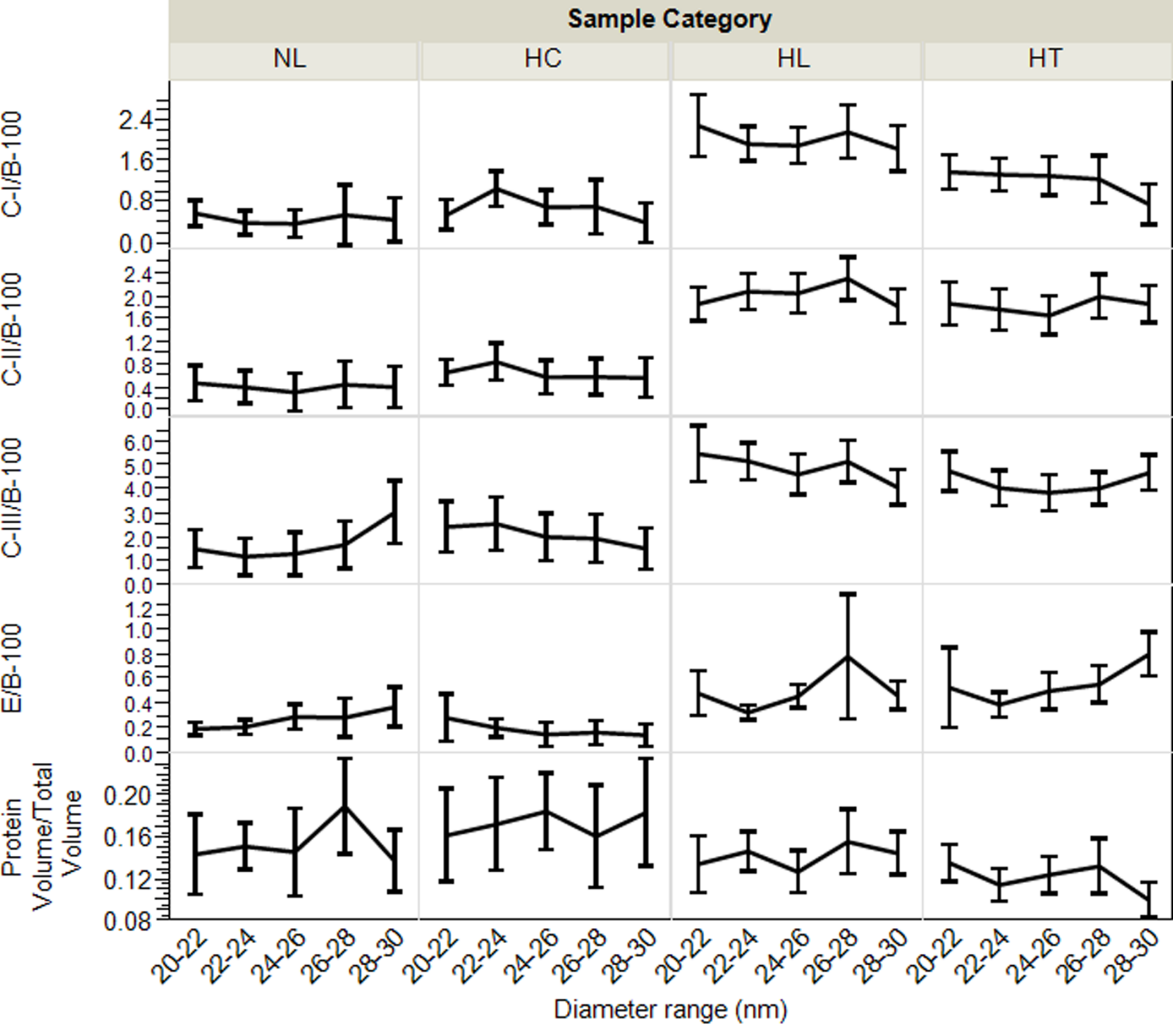
Average exchangeable apo/apoB-100 molar ratio profiles, and protein volume vs. total particle volume ratios by sample categories. Labels indicate normolipidemic (NL, N = 25, Total-C <230 mg/dL, and Total-TG <150 mg/dL); hypercholesterolemic (HC, N = 13, Total-C >230 mg/dL and Total-TG <150 mg/dL); hyperlipidemic (HL, N = 41, Total-C >230 mg/dL and Total-TG >150 mg/dL); and hypertriglyceridemic (HT, N = 31, Total-C<230 mg/dL and Total-TG>150 mg/dL). Error bars indicate confidence intervals.

In the >18 nm fractions, we observed significant differences of the apo and lipid concentration profiles among individual samples (Fig 6), indicating resolution of small, medium and large LDL particles by the AF4 separation. In the 18–30 nm range, the average number of apoC-I, apoC-II, apoC-III and apoE, per Lp-P seemed to gradually increase from 0.5–4, 1–4, 2–9 and 0.25–1 with size, respectively (Fig 9). In the fractions 30–36 nm, the number of apoC-I, apoC-II, apoC-III and apoE per Lp-P increased further, up to around 5, 7, 12 and 2.5, respectively (S9C Fig). However, as mentioned above, in the 18–30 nm range apoB-100/Lp-P also increased on average from 0.8–2.5 (Fig 9), and >30 nm apoB-100/Lp-P was up to >4 (S8 Fig). Consequently, if apoB-100 were used as a measure of Lp-P, the number of apos per apoB-100 molecules were proportionately less and the differences between fractions became also less significant (Fig 11). However, within fractions between sample categories, the average concentration of apoCs and apoE relative to either apoB-100 or Lp-P concentration still differed significantly. Both ApoCs/apoB-100 (Fig 11) and apoCs/Lp-P (S9A Fig) were higher for HL and HT than NL and HC subjects; in spite of the probable bias in the Lp-P calculations discussed above.

Our calculated average values of 1.5–6 apoC-III/apoB-100 and 0.2–0.8 apoE/apoB-100 molar ratios in the LDL fractions (Fig 8) were fairly similar to those derived from reported data, ~2 apoC-III/apoB-100 [51] and ~0.2 apoE/apoB-100 [32]. In these studies, using apoC-III immunocapture followed by ultracentrifugation, 10–25% of the LDL density fraction were found to contain apoC-III, with molar ratios of 16–28 apoC-III/apoB-100 [51] and 1.3–2.4 apoE/apoB-100 [32]. Unfortunately, with our AF4-LC/MS/MS approach the percent of LDL particles that carry apoCs and apoE cannot be elucidated.

### Differences in apo/apo and lipid/lipid molar ratios between sample categories

For the assessment of differences between sample categories we found the calculation of mole/mole ratios of specific analyte pairs most useful: A-I/A-II, C-II/C-I, C-II/C-III, E/C-III, FC/PL, SM/PL, PE/PL, PI/PL and LPC/PL (Fig 9). The AF4 channel recovery of these analyte pairs showed significant correlations; (of r = 0.75, 0.83, 0.76, 0.44, 0.82, 0.86, 0.77, 0.89, and 0.55, respectively, p<0.01). Therefore, these ratios on average within sample categories were independent of Lp-P in the fractions and had minimal corresponding calculation biases.

**Fig 12:**
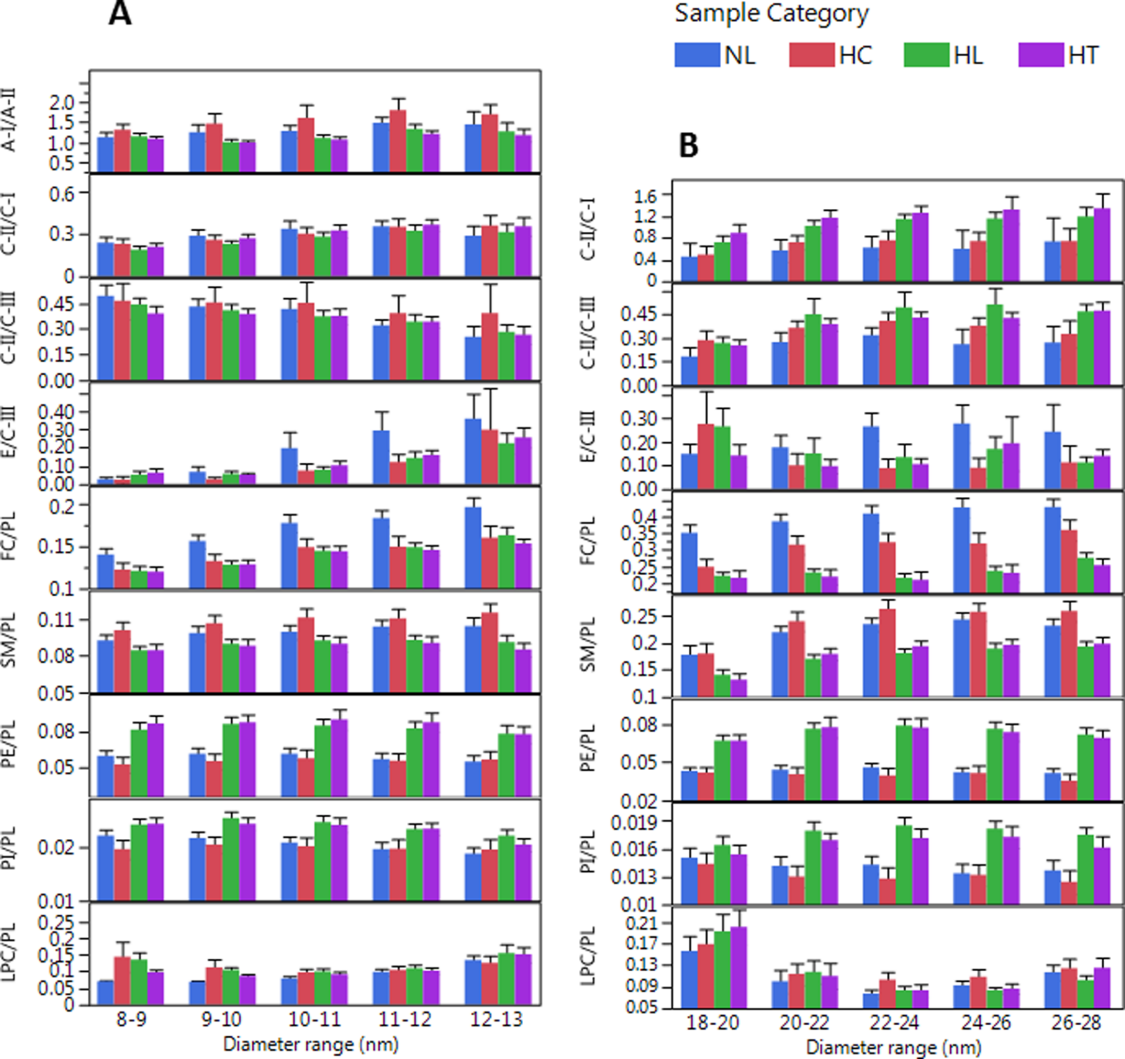
Analyte ratios in size ranges that most characterize sample categories. Error bars indicate confidence intervals (biological variability in sample categories); HDL size region (A), non-HDL size region (B). Labels indicate normolipidemic (NL, N = 25, Total-C <230 mg/dL, and Total-TG <150 mg/dL); hypercholesterolemic (HC, N = 13, Total-C >230 mg/dL and Total-TG <150 mg/dL); hyperlipidemic (HL, N = 41, Total-C >230 mg/dL and Total-TG >150 mg/dL); and hypertriglyceridemic (HT, N = 31, Total-C<230 mg/dL and Total-TG>150 mg/dL).Error bars indicate 95% confidence intervals.

As suggested by previous studies, the E/C-III ratio is a useful measure of the rate of LpB particle uptake; based on evidence that LDL receptor uptake is facilitated by apoE while suppressed by apoC-III [52]. From our data, the E/C-III ratio most significantly differentiated NL from the other sample categories both in the 10–12 nm HDL and in the 22–28 nm LDL size range (Fig 12). The enhanced depletion of LDL particles due to high E/C-III ratio explains the relatively lower apoB-100 levels in NL subjects.

The C-II/C-I and the C-II/C-III ratios are useful for expressing the combined effects of apoCs on the TG depletion rate of LDL and vLDL particles; C-II being a main LPL cofactor, C-I as a CETP inhibitor, and C-III as a LPL inhibitor [51, 53–55]. We found that the C-II/C-I and C-II/C-III ratios in the 20–28 nm LDL fractions significantly differentiated HL and HT from NL subjects (Fig 12). The higher C-II/C-I, C-II/C-III and lower E/C-III ratios are consistent with fast conversion of TG rich VLDL into LDL particles with concurrently slow LDL receptor uptake; and conditions that leads to the accumulation of LDL particles (high apoB-100 levels) in HL and HT subjects.

The A-I/A-II ratios of 1–1.7 in the 8–16 nm size range agreed with previous reports [56, 57]. Because of the ability of apoA-II to displace enzymes and their apo cofactors and inhibitors, ApoA-II indirectly affects LPL and CETP activity and reduces the TG depletion rate of Lp particles [58]. Modeling studies also showed that ApoA-II hinders the expansion of HDL particles to larger size [56]. We found support for these apoA-II functions by observing significantly lower A-I/A-II ratio in HT samples (lower apoA-I/Lp-P vs. apoA-II/Lp-P) in contrast to NL and HC samples with higher A-I/A-II ratio (higher apoA-I/Lp-P relative to apoA-II/Lp-P) (Fig 12). HC samples also exhibited a 1–2 nm shift in the HDL-P maxima to larger size (Fig 6).

The FC/PL, SM/PL, PE/PL, PI/PL and LPC/PL ratios are informative in view of monolayer studies that linked these ratios to physicochemical characteristics of PL monolayers, lateral surface pressure and number of PL molecules per monolayer surface area. For example, higher FC/PL molar ratio results in increased monolayer surface pressure, reduced compressibility and permeability by proteins [59]. Higher SM/PL ratio enhances incorporation of FC molecules into monolayers [60]. The PI/PL and LPC/PL ratios on the surface are important characteristics of Lps due to the role of PI and LPC in various signaling pathways [2]. The PE/PL ratio is informative because PE is a prerequisite for the secretion of VLDL particles [61]. Our AF4-LC-MS/MS approach provides information in vivo by showing significant differences in surface lipid composition between sample categories within HDL and LDL size fractions (Fig 12). Most significantly, the FC/PL ratio was highest in NL, and SM/PL ratio was highest in HC, and the PE/PL and PI/PL ratios were highest in HL and HT samples.

We also performed pairwise correlation analysis among analyte/analyte ratios within size increments without grouping into sample categories. Numerous significant (p<0.05) positive or negative correlations (r = 0.4–0.8) were observed among apo/apo and lipid/lipid ratios (S10 Fig). Both in HDL and LDL size ranges, the FC/PL ratio positively correlated with the SM/PL ratio; there were also positive correlations of SM/PL vs. CE/PL, C-II/C-I vs. C-II/C-III, and TG/PL vs. PE/PL ratios. In the HDL size range, the A-I/A-II ratio correlated positively with C-II/C-III and FC/PL ratios, but negatively with the PE/PL and PI/PL ratios. In the LDL fractions, we observed negative correlation for FC/PL vs. TG/PL; positive correlations for C-II/C-I vs. TG/PL and PE/PL; and positive correlation for C-II/C-III vs. PI/PL. These correlations confirm the ability of our approach to provide metabolic and structural links among Lp constituents and their relevance to Lp functions.

## Conclusion

The main contribution of this work is the demonstration of the efficacy of a multiplexed lipoprotein analysis workflow by analyzing serum samples from 110 persons with wide ranges of Total-C and Total-TG levels. Our workflow provides a comprehensive array of size-specific, lipoprotein composition information relevant to lipid metabolism. Starting with only 100 μL of serum, size fractionation of lipoproteins using asymmetric flow field-flow fractionation and three mass spectrometry methods has provided apolipoprotein and lipid composition information along with lipoprotein particle number. Relative to combination of traditional methods (ultracentrifugation, size exclusion chromatography, gel electrophoresis, immunodiffusion, cross-linking), the presented workflow provides an extensive amount of quantitative lipoprotein composition information for sub-fractions of HDL and LDL correspondingly in one analytical run. We presented results for persons with a wide range of cholesterol and triglycerides levels, showing substantive differences in lipid and protein levels and their ratios. The consistency of our findings with previous studies indirectly validates our AF4-LC-MS/MS approach. The usefulness of these comprehensive lipoprotein composition measurements for characterization of patients with known clinical outcome will be the subject of planned future investigations which may lead to improved prediction of risk for coronary artery disease.

## Supporting information

S1 File LC-MS/MS analysis of non-polar lipids (FC, CE and TG). (DOCX)

S2 File LC-MS/MS analysis of phospholipids. (DOCX)

S3 File LC-MS/MS analysis of apolipoproteins. (DOCX)

S1 Table Summary of repeated analysis of the quality control pool. Showing inter-day mean and %CV for whole serum concentration measurements, and mean±Stddev for the sum of fractions in the indicated size ranges.
(DOCX)

S2 Table Excel table of raw data. Total concentrations in serum, measured hydrodynamic size in fractions, concentrations in fractions, particle number in fractions, analyte/particle, and analyte/analyte ratios.
(XLSX)

S1 Fig MRM chromatogram of non-polar lipids. Free cholesterol (FC), cholesteryl esters (CE) and triglycerides (TG).
(DOCX)

S2 Fig MRM chromatogram of phospholipids. Phosphatidylcholine (PC), sphingomyelin (SM), phosphatidylethanolamine (PE), phosphatidylinositol (PI) and lysophosphatidylcholine (LPC). MRM peak areas of individual component by phospholipid classes (B).
(DOCX)

S3 Fig Overlay of a typical MRM chromatograms of monitored proteolytic peptides. (DOCX)

S4 Fig 96-well plate layout. (DOCX)

S5 Fig Size distribution profile of lipoproteins and enzymes. Detected in AF4 fractions but they were not included into the particle number calculations because of their low concentration in normal serum (<0.2 μM) relative to apoA-I (~50 μM) or apoB (~0.15 μM).
(DOCX)

S6 Fig Comparison of apolipoprotein profiles. In fresh serum (solid lines), after storage at 4°C (A), and at -80°C for 24 hours (B); mean profile from N = 67 measurements after the first thaw during 8 month storage at -80°C (C); mean sum of HDL fractions (D); and sum of LDL fractions (E) by month. Error bars indicate standard deviation.
(DOCX)

S7 Fig AF4 channel recoveries of apoA-I, apoB, PC and TC (CE+FC). Stratified by the range of Total-TG measured in whole serum.
(DOCX)

S8 Fig Calculated apoB-100/Lp-P and other particle characteristics in >30 nm fractions. Error bars indicate standard deviation.
(DOCX)

S9 Fig Comparison of concentration profiles (nmole/L) and molar analyte/Lp-P profiles by sample categories. **A**: Mean size fraction concentrations for apoA-I, apoB-100 and apoE (1st row), and corresponding number of apo/Lp-P molar ratio profiles (below). **B**: Mean size fraction concentrations for apoA-I, apoB-100 and apoE (1st row), mean size fraction concentrations of FC and PL (2nd row), mean fraction concentrations of CE and TG (3^rd^ row), and number of lipid/Lp-P molar ratio profiles (below). **C**: Mean apo/Lp-P molar ratio profiles <30 nm. Error bars indicate confidence intervals.
(DOCX)

S10 Fig Significant (p<0.05) pairwise correlations between molar analyte/analyte ratios by size increments. A: 8–13 nm; B: 14–20 nm; C: >20 nm.
(DOCX)

## Abbreviation

Lp: Lipoproteins
Lp-P: lipoprotein particle number
apoA-I: apolipoprotein A-I
apoA-II: apolipoprotein A-II
apoA-IV: apolipoprotein A-IV
apoC-I: apolipoprotein C-I
apoC-II: apolipoprotein C-II
apoC-III: apolipoprotein C-III
apoE: apolipoprotein E
LpA: apolipoprotein A-1 containing particles
LpB: apolipoprotein B containing particles
PL: phospholipids
Total-C: total cholesterol
FC: free cholesterol
CE: cholesterol esters
TG: triglycerides
PL: phospholipids
PC: phosphatidylcholine
SM: sphingomyelin
PE: phosphatidylethanolamine
PI: phosphatidylinositol
LPC: lysophosphatidylcholine
CETP: cholesterylester transfer protein
AF4: asymmetric flow field-flow fractionation
MS: mass spectrometry
LC: liquid chromatography
CV: coefficient of variation
