# Supplementary material for: Core lipid, surface lipid and apolipoprotein composition analysis of lipoprotein particles as a function of particle size in one workflow integrating asymmetric flow field-flow fractionation and liquid chromatography-tandem mass spectrometry
Source: PLoS One. 2018 Apr 10;13(4):e0194797. doi: 10.1371/journal.pone.0194797 (PMC5892890; doi:10.1371/journal.pone.0194797)
Supplement: S1 File — (DOCX) [file pone.0194797.s001.docx]

## **S1 File: LC-MS/MS analysis of non-polar lipids (FC, CE and TG)**

Known representative mixtures of CE and TG were used as calibrators with stable isotope labeled analogs as internal standards. A 50 μL aliquot of each AF4 fraction, or 1:100 diluted serum in AF4 buffer, was placed on a 96-well plate, along with 50 μL aliquots of the calibrator dilution series. To all wells, a 200 µL mix of the IS in EtOH was added. The plate was vortex-mixed on an orbital shaker at 500 rpm for 2 minutes. Samples were evaporated and reconstituted in 50µL of nonane and mixed on an orbital shaker at 500 rpm for 2 minutes. The plate was then sealed with a heat activated aluminum foil cover, and centrifuged for 3 min at 3700 rpm. From each sample supernatant (insoluble pellet stayed at bottom of the well), 6µL was injected into the UHPLC system (details are provided in Supplementary Information). From each extract, 6µL was injected into the Agilent 1290 UHPLC system. The column used was a Kinetex HILIC 1.7µm, 2.1x50mm (Phenomenex, Torrance, CA). Mobile phase A was hexanes with 0.05% isopropanol. Mobile phase B was hexanes with 5% ethanol and 0.05% isopropanol. The mobile phase flow rate was 600 µL/min. The total run time was 2.5 min. The Sciex 4000 QTrap mass spectrometer (Sciex, Framingham, MA) was operated in APCI mode with nitrogen nebulizer gas, at 450 ºC with optimal cone voltage to achieve in-source collision induced dissociation (CID) fragmentation of the CEs and TGs. The CE and TG analyte group specific precursor ion fragments were further fragmented in multiple reaction monitoring mode (FC and CE: 369🡪161 m/z, TG: 95🡪67 m/z). Typical multiple reaction monitoring (MRM) chromatograms are shown in Supporting Information (Figure S2).
