# Supplementary material for: Core lipid, surface lipid and apolipoprotein composition analysis of lipoprotein particles as a function of particle size in one workflow integrating asymmetric flow field-flow fractionation and liquid chromatography-tandem mass spectrometry
Source: PLoS One. 2018 Apr 10;13(4):e0194797. doi: 10.1371/journal.pone.0194797 (PMC5892890; doi:10.1371/journal.pone.0194797)
Supplement: S2 File — (DOCX) [file pone.0194797.s002.docx]

## **S2 File: LC-MS/MS analysis of phospholipids**

Quantification of phosphatidylcholine (PC), sphingomyelin (SM), phosphatidylethanolamine (PE), phosphatidylinositol (PI), and lysophosphatidylcholine (LPC) was performed using a calibrator serum pool, value assigned using representative mix of PL standards from Avanti Polar Lipids (Alabaster, AL, USA). To a 20 µL aliquot of each AF4 fraction, diluted serum or calibration standard, a 200 µL mix of the IS in EtOH was added. The plate was vortex-mixed on an orbital shaker at 500 rpm for 2 minutes. Samples were evaporated until dryness, reconstituted with 50 μL nonane/i-PrOH/water, and the plate was mixed on an orbital shaker at 500 rpm for 2 minutes; then the plate was sealed with a heat activated aluminum foil cover, and centrifuged for 3 min at 3700 rpm. From each sample supernatant (insoluble pellet stayed at bottom of the well), 5µL was injected into the LC-MS/MS system. From each extract, 5µL was injected into the Acquity UHPLC system (Waters, USA) equipped with a Kinetex HILIC 1.7µm, 2.1x100mm , 3µm particle column. The separation was performed in acetonitrile/water/ isopropanol solvent gradient with 0.7 mL/min flow rate, 4 min per injection. A 6500 Qtrap (Sciex, Framingham, MA) was operated in MRM scanning mode, monitoring approximately 10-20 of the typically most abundant PE, PI, PC, LPC and SM transitions. The calibration curve was constructed with the sum of all transitions for each PL class. A typical total ion chromatogram is shown in Supporting Information (Figure S3).
