# Supplementary material for: Core lipid, surface lipid and apolipoprotein composition analysis of lipoprotein particles as a function of particle size in one workflow integrating asymmetric flow field-flow fractionation and liquid chromatography-tandem mass spectrometry
Source: PLoS One. 2018 Apr 10;13(4):e0194797. doi: 10.1371/journal.pone.0194797 (PMC5892890; doi:10.1371/journal.pone.0194797)
Supplement: S3 File — (DOCX) [file pone.0194797.s003.docx]

## **S3 File: LC-MS/MS analysis of apolipoproteins**

Apos A-I, A-II, A-IV, B-100, C-I, C-II, C-III and E in each fraction and in 1:100 diluted serum were measured using a dilution series of a characterized serum pool as calibrator. The A-II, A-IV, C-I, C-II, C-III and E content of the calibrator serum pool was value assigned using standard addition of known concentration of pure recombinant protein standards. For apos A-I and B-100 the calibrator pool was analyzed by an in-solution digestion method based on synthetic peptide calibrators (34). To assure calibration reproducibility, after value assignment, different amounts of the calibrator pool were pre-aliquoted and weighed before storing at -80 ⁰C. Before use, the stored known amount of aliquots were filled up to 1 mL with AF4 buffer.

To each well (including calibrators and fractions, along with 1:100 diluted LCMS-QC, unknown serum aliquots, and AF4-QC serum), 50 μL 0.45% Zwittergent 3-12 in digest buffer (2 mM CaCl_2_, 50 mM TRIS, pH 8.4) was added to a nominal concentration of 0.15% in the well. The sample plate was mixed on a plate shaker at 500 rpm for 2 min and placed directly into the autosampler kept at 8°C. A typical batch from AF4 fractionation of 5 unknown and 1 AF4-QC samples (in 3 96-well plates) was analyzed on 3 separate LC-MS/MS workstations. Each workstation included an integrated on-line trypsin digestion coupled LC system (Perfinity Biosciences, USA) and a mass spectrometer (6500 QTRAP®, Sciex, Framingham, MA). The sample injection sequence started by drawing 5 μL of IS peptide solution from a reagent vial into the sample loop, immediately followed by drawing 50 μL sample from the 96-well plate. Assuming that a typical human serum sample contains 60-80 g/L of protein, with injection of 50 µL of 1500x-16x diluted calibrator serum pool, the maximum total protein amount injected was between 2-250 µg.

The Perfinity trypsin column (2.1 mm x 33 mm) was operated at 50°C with a 25 µL/min flow rate, giving an injection-plug digestion/breakthrough time of 3-4 minutes. The unreacted proteins, native cleavage products and internal standards were carried together directly to the trapping column (Halo® C18 4.6 mm x 5 mm, 2.7 µm guard column). After the digestion/trapping period, native cleavage products and IS peptides were eluted from the trapping column to the analytical column (Halo® C18 core shell HPLC column, 2.1 mm x 100 mm, 2.7 µm). The analytical separation was performed at 50°C with a flow rate of 350 µL/min over a stepwise gradient ranging from 3% to 95% acetonitrile containing 0.1% formic acid. The native and the isotopically labeled IS peptide chromatograms were acquired by multiple reaction monitoring (MRM) with unit mass resolution, in scheduled 60 s acquisition windows with a 0.65 s target scan time. Typical MRM chromatograms of a 1:100 diluted LCMS-QC serum pool are shown in Supporting Information (Figure S4). The column switching valve design and addition of a second trapping column allowed simultaneous digestion/trapping and LC-MS/MS analysis, increasing instrument throughput to 12.5 min per injection.
