# Supplementary material for: Core lipid, surface lipid and apolipoprotein composition analysis of lipoprotein particles as a function of particle size in one workflow integrating asymmetric flow field-flow fractionation and liquid chromatography-tandem mass spectrometry
Source: PLoS One. 2018 Apr 10;13(4):e0194797. doi: 10.1371/journal.pone.0194797 (PMC5892890; doi:10.1371/journal.pone.0194797)
Supplement: S1 Table — Showing inter-day mean and %CV for whole serum concentration measurements, and mean±Stddev for the sum of fractions in the indicated size ranges. (DOCX) [file pone.0194797.s004.docx]

**S1 Table. Summary of repeated analysis of the quality control pool.** Showing inter-day mean and %CV for whole serum concentration measurements, and mean±Stddev for the sum of fractions in the indicated size ranges.

|  | Whole serum | | | Albumin | HDL | | | LDL | | r-Lp |
| --- | --- | --- | --- | --- | --- | --- | --- | --- | --- | --- |
|  |  |  |  | <7 nm | 7-10 nm | 10-13 nm | 13-18 nm | 18-23 nm | 23-30 nm | >30 nm |
| Analyte | Mean | Intra-run  %CV  (N=3) | Inter-day  %CV  (N=75) | Mean±Stddev (Inter-day, N=25) | | | | | | |
|  |  |  |  |  |  |  |  |  |  |  |
| *Non-polar lipids (µM)* | | | | | | | | | | |
| FC | 1148 | 5% | 7% | 12.1 (4) | 170 (17.1) | 101.5 (19.9) | 36.6 (15.3) | 322.5 (101.2) | 372.7 (52.8) | 135.5 (32.1) |
| CE | 3775 | 6% | 9% | 24.7 (2.2) | 853.9 (98.4) | 462.3 (109.5) | 110.2 (44) | 973.3 (205.9) | 1049.7 (230) | 305.3 (53.9) |
| TG | 1050 | 6% | 10% | 51.4 (4.4) | 215.3 (29.8) | 118.9 (47.8) | 54.1 (43.3) | 202.4 (19.5) | 269.4 (76.4) | 194.9 (50) |
| *Polar lipids (µM)* | | | | | | | | | | |
| PC | 2176 | 4% | 7% | 45.7 (8.6) | 838.3 (99.5) | 399.7 (58.3) | 91.9 (30.4) | 339 (65.5) | 357.4 (78.1) | 137.2 (33.7) |
| SM | 415 | 4% | 6% | 4.9 (1.7) | 77 (8.9) | 40.4 (6.1) | 12.2 (4.8) | 112.5 (21.3) | 127.4 (30) | 45 (7) |
| PE | 137 | 3% | 11% | 2.7 (0.6) | 52.6 (4.9) | 27 (5.4) | 6 (1.6) | 21.2 (6.2) | 21.2 (3.2) | 8.2 (1.3) |
| PI | 62 | 2% | 5% | 1.4 (0.3) | 26 (2.9) | 11.8 (2.8) | 2.5 (0.9) | 8.5 (1.7) | 8.8 (1.8) | 3.1 (0.7) |
| LPC | 517 | 4% | 6% | 142.8 (37.6) | 224 (119.1) | 43.8 (9.9) | 29.5 (11.3) | 44 (12.7) | 51.2 (12.4) | 34.8 (10.6) |
| *Apolipoproteins (µM)* | | | | | | | | | | |
| apoA-I | 44.34 | 3% | 12% | 0.96 (0.38) | 30.44 (4.17) | 12.17 (2.08) | 1.12 (0.73) |  |  |  |
| apoA-II | 49.31 | 5% | 12% | 0.92 (1.12) | 37.71 (8.02) | 9.24 (2.76) | 0.21 (0.21) |  |  |  |
| apoA-IV | 1.83 | 3% | 13% | 0.14 (0.04) | 1.62 (0.23) | 0.12 (0.09) |  |  |  |  |
| apoB-100 | 1.68 | 6% | 13% |  |  |  |  | 0.7 (0.11) | 0.79 (0.22) | 0.16 (0.06) |
| apoC-I | 11.43 | 5% | 19% |  | 6.28 (0.86) | 3.56 (0.93) | 0.44 (0.36) | 0.47 (0.14) | 0.51 (0.21) | 0.02 (0.03) |
| apoC-II | 3.28 | 4% | 15% |  | 1.34 (0.24) | 1.11 (0.3) | 0.1 (0.07) | 0.27 (0.12) | 0.34 (0.14) | 0.07 (0.08) |
| apoC-III | 9.05 | 5% | 16% |  | 3.43 (0.87) | 3.41 (0.72) | 0.46 (0.29) | 0.79 (0.33) | 0.87 (0.5) | 0 (0) |
| apoE | 1.66 | 11% | 25% |  | 0.23 (0.07) | 0.63 (0.1) | 0.36 (0.07) | 0.11 (0.05) | 0.19 (0.05) | 0.09 (0.03) |
