# Supplementary material for: Core lipid, surface lipid and apolipoprotein composition analysis of lipoprotein particles as a function of particle size in one workflow integrating asymmetric flow field-flow fractionation and liquid chromatography-tandem mass spectrometry
Source: PLoS One. 2018 Apr 10;13(4):e0194797. doi: 10.1371/journal.pone.0194797 (PMC5892890; doi:10.1371/journal.pone.0194797)
Supplement: S1 Fig — Free cholesterol (FC), cholesteryl esters (CE) and triglycerides (TG). (DOCX) [file pone.0194797.s006.docx]

**S1 Fig**. **MRM chromatogram of non-polar lipids.** Free cholesterol (FC), cholesteryl esters (CE) and triglycerides (TG).
