# Supplementary material for: Core lipid, surface lipid and apolipoprotein composition analysis of lipoprotein particles as a function of particle size in one workflow integrating asymmetric flow field-flow fractionation and liquid chromatography-tandem mass spectrometry
Source: PLoS One. 2018 Apr 10;13(4):e0194797. doi: 10.1371/journal.pone.0194797 (PMC5892890; doi:10.1371/journal.pone.0194797)
Supplement: S2 Fig — Phosphatidylcholine (PC), sphingomyelin (SM), phosphatidylethanolamine (PE), phosphatidylinositol (PI) and lysophosphatidylcholine (LPC). MRM peak areas of individual component by phospholipid classes (B). (DOCX) [file pone.0194797.s007.docx]

**S2 Fig**. **MRM chromatogram of phospholipids**. Phosphatidylcholine (PC), sphingomyelin (SM), phosphatidylethanolamine (PE), phosphatidylinositol (PI) and lysophosphatidylcholine (LPC). MRM peak areas of individual component by phospholipid classes (B).

A

B
