# Supplementary material for: Core lipid, surface lipid and apolipoprotein composition analysis of lipoprotein particles as a function of particle size in one workflow integrating asymmetric flow field-flow fractionation and liquid chromatography-tandem mass spectrometry
Source: PLoS One. 2018 Apr 10;13(4):e0194797. doi: 10.1371/journal.pone.0194797 (PMC5892890; doi:10.1371/journal.pone.0194797)
Supplement: S5 Fig — Detected in AF4 fractions but they were not included into the particle number calculations because of their low concentration in normal serum (<0.2 μM) relative to apoA-I (~50 μM) or apoB (~0.15 μM). (DOCX) [file pone.0194797.s010.docx]

**S5 Fig**. **Size distribution profile of lipoproteins and enzymes**. Detected in AF4 fractions but they were not included into the particle number calculations because of their low concentration in normal serum (<0.2 µM) relative to apoA-I (~50 µM) or apoB (~0.15 µM).
