# Supplementary material for: Core lipid, surface lipid and apolipoprotein composition analysis of lipoprotein particles as a function of particle size in one workflow integrating asymmetric flow field-flow fractionation and liquid chromatography-tandem mass spectrometry
Source: PLoS One. 2018 Apr 10;13(4):e0194797. doi: 10.1371/journal.pone.0194797 (PMC5892890; doi:10.1371/journal.pone.0194797)
Supplement: S6 Fig — In fresh serum (solid lines), after storage at 4°C (A), and at -80°C for 24 hours (B); mean profile from N = 67 measurements after the first thaw during 8 month storage at -80°C (C); mean sum of HDL fractions (D); and sum of LDL fractions (E) by month. Error bars indicate standard deviation. (DOCX) [file pone.0194797.s011.docx]

**S6 Fig**. **Comparison of apolipoprotein profiles**. In fresh serum (solid lines), after storage at 4 °C (A), and at -80 °C for 24 hours (B); mean profile from N=67 measurements after the first thaw during 8 month storage at -80 °C (C); mean sum of HDL fractions (D); and sum of LDL fractions (E) by month. Error bars indicate standard deviation.

**S6 Fig** cont.
