# Supplementary material for: Core lipid, surface lipid and apolipoprotein composition analysis of lipoprotein particles as a function of particle size in one workflow integrating asymmetric flow field-flow fractionation and liquid chromatography-tandem mass spectrometry
Source: PLoS One. 2018 Apr 10;13(4):e0194797. doi: 10.1371/journal.pone.0194797 (PMC5892890; doi:10.1371/journal.pone.0194797)
Supplement: S7 Fig — Stratified by the range of Total-TG measured in whole serum. (DOCX) [file pone.0194797.s012.docx]

**S7 Fig. AF4 channel recoveries of apoA-I, apoB, PC and TC (CE+FC)**. Stratified by the range of Total-TG measured in whole serum.
