# Supplementary material for: Core lipid, surface lipid and apolipoprotein composition analysis of lipoprotein particles as a function of particle size in one workflow integrating asymmetric flow field-flow fractionation and liquid chromatography-tandem mass spectrometry
Source: PLoS One. 2018 Apr 10;13(4):e0194797. doi: 10.1371/journal.pone.0194797 (PMC5892890; doi:10.1371/journal.pone.0194797)
Supplement: S8 Fig — Error bars indicate standard deviation. (DOCX) [file pone.0194797.s013.docx]

**S8 Fig**. **Calculated apoB-100/Lp-P and other particle characteristics in >30 nm fractions.** Error bars indicate standard deviation.

>40
