# Supplementary material for: Core lipid, surface lipid and apolipoprotein composition analysis of lipoprotein particles as a function of particle size in one workflow integrating asymmetric flow field-flow fractionation and liquid chromatography-tandem mass spectrometry
Source: PLoS One. 2018 Apr 10;13(4):e0194797. doi: 10.1371/journal.pone.0194797 (PMC5892890; doi:10.1371/journal.pone.0194797)
Supplement: S9 Fig — A: Mean size fraction concentrations for apoA-I, apoB-100 and apoE (1st row), and corresponding number of apo/Lp-P molar ratio profiles (below). B: Mean size fraction concentrations for apoA-I, apoB-100 and apoE (1st row), mean size fraction concentrations of FC and PL (2nd row), mean fraction concentrations of CE and TG (3rd row), and number of lipid/Lp-P molar ratio profiles (below). C: Mean apo/Lp-P molar ratio profiles <30 nm. Error bars indicate confidence intervals. (DOCX) [file pone.0194797.s014.docx]

**S9 Fig. Comparison of concentration profiles (nmol/L) and molar analyte/Lp-P profiles by sample categories.** **A**: Mean size fraction concentrations for apoA-I, apoB-100 and apoE (1st row), and corresponding number of apo/Lp-P molar ratio profiles (below). **B**: Mean size fraction concentrations for apoA-I, apoB-100 and apoE (1st row), mean size fraction concentrations of FC and PL (2nd row), mean fraction concentrations of CE and TG (3^rd^ row), and number of lipid/Lp-P molar ratio profiles (below). **C**: Mean apo/Lp-P molar ratio profiles <30 nm. Error bars indicate confidence intervals.

**A**

**S9 Fig cont.**

**B**

**&ApoB-100**

**Supporting Figure S9 cont.**

**C**
