# Supplementary material for: Core lipid, surface lipid and apolipoprotein composition analysis of lipoprotein particles as a function of particle size in one workflow integrating asymmetric flow field-flow fractionation and liquid chromatography-tandem mass spectrometry
Source: PLoS One. 2018 Apr 10;13(4):e0194797. doi: 10.1371/journal.pone.0194797 (PMC5892890; doi:10.1371/journal.pone.0194797)
Supplement: S10 Fig — A: 8–13 nm; B: 14–20 nm; C: >20 nm. (DOCX) [file pone.0194797.s015.docx]

**S10 Fig.** **Significant (p<0.05) pairwise correlations between molar analyte/analyte ratios by size increments.** A: 8-13 nm; B: 14-20 nm; C: >20 nm

A

**S10 Fig cont.**

**B**

**S10 Fig cont.**

**C**
